# Quantifying the impact of novel metastatic cancer therapies on health inequalities in survival outcomes

**DOI:** 10.3389/fphar.2023.1249998

**Published:** 2023-11-24

**Authors:** Karolina Zebrowska, Rosa C. Banuelos, Evelyn J. Rizzo, Kathy W. Belk, Gary Schneider, Koen Degeling

**Affiliations:** ^1^ Healthcare Consultancy Group, London, United Kingdom; ^2^ Healthcare Consultancy Group, New York, NY, United States

**Keywords:** oncology, inequality, overall survival, progression-free survival, health disparities, colorectal cancer, non-small cell lung cancer, breast cancer

## Abstract

**Background:** Novel therapies in metastatic cancers have contributed to improvements in survival outcomes, yet real-world data suggest that improvements may be mainly driven by those patient groups who already had the highest survival outcomes. This study aimed to develop and apply a framework for quantifying the impact of novel metastatic cancer therapies on health inequalities in survival outcomes based on published aggregate data.

**Methods:** Nine (N = 9) novel therapies for metastatic breast cancer (mBC), metastatic colorectal cancer (mCRC), and metastatic non–small cell lung cancer (mNSCLC) were identified, 3 for each cancer type. Individual patient data (IPD) for overall survival (OS) and progression-free survival (PFS) were replicated from published Kaplan-Meier (KM) curves. For each cancer type, data were pooled for the novel therapies and comparators separately and weighted based on sample size to ensure equal contribution of each therapy in the analyses. Parametric (mixture) distributions were fitted to the weighted data to model and extrapolate survival. The inequality in survival was defined by the absolute difference between groups with the highest and lowest survival for 2 stratifications: one for which survival was stratified into 2 groups and one using 5 groups. Additionally, a linear regression model was fitted to survival estimates for the 5 groups, with the regression coefficient or slope considered as the inequality gradient (IG). The impact of the pooled novel therapies was subsequently defined as the change in survival inequality relative to the pooled comparator therapies. A probabilistic analysis was performed to quantify parameter uncertainty.

**Results:** The analyses found that novel therapies were associated with significant increases in inequalities in survival outcomes relative to their comparators, except in terms of OS for mNSCLC. For mBC, the inequalities in OS increased by 13.9 (95% CI: 1.4; 26.6) months, or 25.0%, if OS was stratified in 5 groups. The IG for mBC increased by 3.2 (0.3; 6.1) months, or 24.7%. For mCRC, inequalities increased by 6.7 (3.0; 10.5) months, or 40.4%, for stratification based on 5 groups; the IG increased by 1.6 (0.7; 2.4) months, or 40.2%. For mNSCLC, inequalities decreased by 14.9 (−84.5; 19.0) months, or 12.2%, for the 5-group stratification; the IG decreased by 2.0 (−16.1; 5.1) months, or 5.5%. Results for the stratification based on 2 groups demonstrated significant increases in OS inequality for all cancer types. In terms of PFS, the increases in survival inequalities were larger in a relative sense compared with OS. For mBC, PFS inequalities increased by 8.7 (5.9; 11.6) months, or 71.7%, for stratification based on 5 groups; the IG increased by 2.0 (1.3; 2.6) months, or 67.6%. For mCRC, PFS inequalities increased by 5.4 (4.2; 6.6) months, or 147.6%, for the same stratification. The IG increased by 1.3 (1.1; 1.6) months, or 172.7%. For mNSCLC, inequalities increased by 18.2 (12.5; 24.4) months, or 93.8%, for the 5-group stratification; the IG increased by 4.0 (2.8; 5.4) months, or 88.1%. Results from the stratification based on 2 groups were similar.

**Conclusion:** Novel therapies for mBC, mCRC, and mNSCLC are generally associated with significant increases in survival inequalities relative to their comparators in randomized controlled trials, though inequalities in OS for mNSCLC decreased nonsignificantly when stratified based on 5 groups. Although further research using real-world IPD is warranted to assess how, for example, social determinants of health affect the impact of therapies on health inequalities among patient groups, the proposed framework can provide important insights in the absence of such data.

## Introduction

Disparities and inequalities in cancer survival outcomes exist, and they have been well-documented in equity-informed literature. Studies that examine survival disparities in patients undergoing oncology care have found that treatment improved overall survival (OS); however, social determinants of health (SDOH), such as Black race, low income, lack of insurance, and low educational attainment, have been associated with poorer OS outcomes (Acharya et al., 2016; Austin et al., 2016; Cui and Finkelstein, 2022; Fabregas et al., 2022; Lee and Singh, 2022; Namburi et al., 2022; Tran et al., 2022; Alnajar et al., 2023). For example, one study found that the percentage of individuals with survival <1 year after diagnosis in Black individuals and White individuals was 41.4% and 22.2% for lung cancer, 9.8% and 7.1% for colorectal cancer, and 2.9% and 0.7% for breast cancer, respectively (Cui and Finkelstein, 2022). Another study showed that patients with advanced lung cancer living in the most materially deprived areas had the shortest median survival time (Qureshi et al., 2023). Hamers et al. (2020) found that, out of all patients diagnosed with stage IV colorectal cancer between 2008 and 2016 in the Netherlands Cancer Registry, OS improved only for those patients who were already doing well compared with others. Further, Asaria et al. (2015) demonstrated that, compared with no screening, a UK bowel cancer screening program improved health across the distribution but widened health inequality between the healthiest and least healthy participants.

There are several methodologic approaches to quantifying inequalities within healthcare from a health economic perspective. These include distributional cost-effectiveness analysis, extended cost-effectiveness analysis, equity-based weighting, multiple criteria decision analysis, and mathematical programming (Ward et al., 2022). A challenge in the use of these methods is that they are mostly informed by granular patient-level data on the relationship between SDOH and health outcomes. SDOH operate at individual, community, and population levels to impact health outcomes (Sengupta and Honey, 2020) and include but are not limited to socioeconomic factors, clinical factors, behavioral factors, environmental factors, and biological factors (American Association for Cancer Research, 2022). However, such data are often not available, which limits the feasibility of performing these types of equity-informed health economic analyses.

To facilitate equity-informed analyses in the absence of individual patient data (IPD), this study aimed to develop and apply a framework for quantifying the impact of novel metastatic cancer therapies on health inequalities in survival outcomes based on aggregate data. The framework was applied to estimate the impact of novel therapies on OS and progression-free survival (PFS) outcomes in metastatic breast cancer (mBC), metastatic colorectal cancer (mCRC), and metastatic non–small cell lung cancer (mNSCLC).

## Materials and methods

### Framework

**FIGURE 1:**
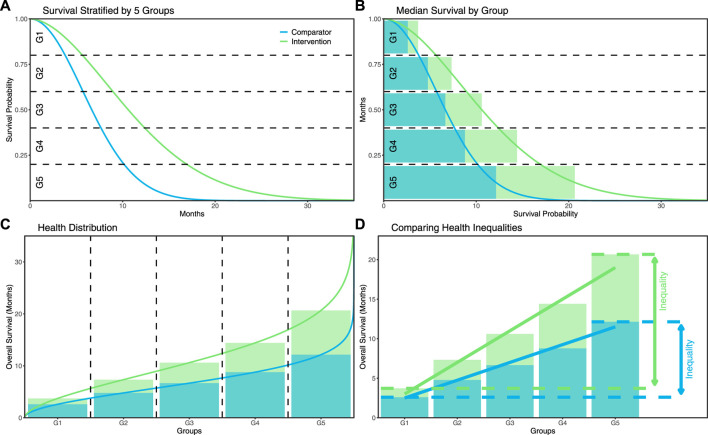
**(A)** illustration of the definition of the patient groups for the 5-group stratification of survival; **(B)** illustration of the median survival in each group; **(C)** illustration of the resulting health distribution based on the median survival in each group; **(D)** illustration of the definition of the health inequalities.

The proposed framework defines the distribution of health in terms of survival of the different patient groups that can be stratified in Kaplan-Meier (KM) curves. This analysis focuses on 2 stratifications: a distribution based on 2 groups and a distribution based on 5 groups of survival. Although the number of groups in which survival will be stratified is a somewhat arbitrary choice, the 5-group stratification was used here because this number of groups is often used to define distributions across populations, for example, based on socioeconomic quintiles (Cookson et al., 2017). The 2-group stratification was additionally applied to investigate and demonstrate that results may change when a different number of groups is used, and to illustrate that even this most basic stratification can provide meaningful insights. Figure 1A illustrates the stratification based on 5 groups. The distribution of health can subsequently be obtained from the median survival within each group, as illustrated in Figure 1B, C. Given that most survival data are censored, this step may involve parametric survival modeling to extrapolate survival curves.

For both the 2-group and 5-group stratifications, the inequality in survival for a certain treatment was defined by the absolute difference in survival between the highest and lowest groups. This is shown visually in Figure 1D. To consider the health distribution across all patient groups for the 5-group stratification, the survival inequality was additionally defined based on the regression slope of a simple linear regression model fitted to the outcomes of all 5 groups, referred to as the inequality gradient (IG).

The impact of novel therapies on survival inequalities can then be defined by the absolute and relative change in the survival inequality (i.e., absolute difference in survival between the lowest and highest groups and the IG) relative to their comparators.

### Application of the framework

To quantify the impact of novel metastatic cancer therapies on health inequalities through the above framework, it was applied to novel therapies for mBC, mCRC, and mNSCLC. These metastatic cancer types were selected based on their incidence and the availability of novel therapies that met the inclusion criteria. For each cancer type, 3 novel drugs were identified based on the following 5 criteria: a) US Food and Drug Administration drug approval between January 2015 and January 2023; b) availability of results from a Phase III randomized controlled trial (RCT); c) at least 100 patients in each arm of the RCT; d) published KM curves for OS and PFS; and e) sufficient follow-up such that the OS and PFS were lower than 35% at the end of follow-up, to reduce the impact of structural assumptions in any survival extrapolations. Table 1 provides an overview of the novel therapies that were selected and their comparators.

**TABLE 1:** Intervention and comparator combinations included in the analysis.

Novel therapy (intervention)	Comparator	Clinical trial	**References**
mBC			
Neratinib + capecitabine	Lapatinib + capecitabine	NALA	Saura et al. (2020)
Tucatinib, trastuzumab, and capecitabine	Placebo, trastuzumab, and capecitabine	HER2CLIMB	Curigliano et al. (2022)
Margetuximab + chemotherapy	Trastuzumab + chemotherapy	SOPHIA	Rugo et al. (2021)
mCRC			
Encorafenib + cetuximab + binimetinib	Investigator’s choice - either cetuximab + irinotecan or cetuximab + FOLFIRI (control)	BEACON	Tabernero et al. (2021)
Regorafenib	Placebo	CORRECT	Grothey et al. (2013)
Trifluridine/tipiracil	Placebo	TERRA	Xu et al. (2018)
mNSCLC			
Osimertinib^a^	First-generation or second-generation EGFR-TKI	FLAURA	PFS: (Soria et al., 2018)
			OS: (Ramalingam et al., 2020)
Nivolumab	Docetaxel	CHECKMATE 078	Wu et al. (2019)
Pembrolizumab^b^ + ipilimumab	Chemotherapy	KEYNOTE-042	Mok et al. (2019)

^a^ Although Soria et al. (2018) included OS, data, the follow-up period was not sufficient (35% survival not reached), hence Ramalingam et al. (2020) was used.

^b^ PD-L1 TPS, of >50%.

For each treatment, IPD for OS and PFS were replicated from the KM curves and summary statistics using the method by Guyot et al. (2012). Beyond visual inspection, the replication process was validated by analyzing the replicated IPD and comparing the results with those reported in the corresponding publications. Subsequently, for each cancer type separately, the IPD for the 3 novel therapies were pooled, as were the data for the 3 comparators, with weighting applied based on the corresponding sample sizes such that each therapy contributed equally to the analysis. Although the framework can be applied to evaluate the impact of specific drugs, data of multiple interventions were pooled because the purpose of this work was to illustrate the proposed framework and not to perform such head-to-head comparisons. The studies used in this analysis used a common criterion, namely, the RECIST v1.1, for OS and PFS definitions. This allowed for straightforward aggregation of individual studies. It must be noted that this is not always the case, and caution must be exercised when pooling data derived from studies that use different criteria for survival outcomes.

Parametric survival modeling was performed to obtain the complete survival distributions for the pooled sets of novel therapies and comparators. Standard parametric distributions and mixtures of 2 distributions were explored, considering the following distributions: exponential, Gamma, Gompertz, log-logistic, log-normal, and Weibull (Gray et al., 2021). Relative modeling of the treatment effects, for example, through parameterization of the distributions’ scale/rate parameter as hazard ratio, was not considered because that would result in increased survival inequalities by definition. More specifically, applying a single relative effect for the interventions compared with the comparators will result in larger absolute change for groups with a higher baseline and, hence, increase inequalities. To reduce the potential impact of structural uncertainty on the outcomes, the same type of distribution was used for the pooled novel therapies and their comparators for each cancer-outcome combination. As the selection of an inappropriate survival model can strongly bias survival estimates and lead to inaccurate results (Gray et al., 2021), an algorithm was defined to select the survival distribution used in the analyses. First, 10-year relative survival rates from the Surveillance, Epidemiology, and End Results (SEER) database were used to define an upper threshold for survival extrapolations (14.8% for mBC (National Cancer Institute Surveillance, Epidemiology, and End Results Program, 2022a), 10% for mCRC (National Cancer Institute Surveillance, Epidemiology, and End Results Program, 2022b), and 3.3% for mNSCLC (National Cancer Institute Surveillance, Epidemiology, and End Results Program, 2022c)). This study allowed for a 10% relative increase of these survival rates to account for novel therapies that may increase survival compared with the treatments used during the SEER data-capture period. Second, the survival distributions for which both the pooled novel therapies and comparators did not exceed the extrapolation threshold were ranked based on their combined Akaike information criterion (AIC) for each distribution type, where a lower AIC indicated a better fit. Finally, the survival distribution with the lowest AIC was selected after a visual inspection to ensure that it was realistic and did not substantially underestimate survival, for example. See the Supplementary Material for an illustration of the selection algorithm and the results of its application for the different cancer types and outcomes. 

### Analyses and availability of material

All results were generated through a probabilistic analysis to quantify the impact of parameter uncertainty on the outcomes and the uncertainty around those outcomes. Multivariate normal distributions were used to define the uncertainty in the survival model parameters. All analyses were performed in R version 4.2.2, and a simplified example of the code used in this analysis has been made available in the following GitHub repository: https://github.com/koendegeling/Survival_Inequalities. The flexsurv package, version 2.2.1 (Jackson, 2016), was used for standard parametric survival modeling.

## Results

Inequalities in OS and PFS significantly increased when comparing the combined novel therapies with their comparators in RCTs, except for mNSCLC, where there was a nonsignificant decrease in OS inequality for the 5-group stratification. The full results are presented by outcome in the following 2 subsections. Tables 2, 3 show the full results of OS and PFS, respectively.

**TABLE 2:** Survival inequalities in terms of OS for all cancers, reported as mean (95% confidence interval) based on the probabilistic analysis.

	2-Group stratification			5-Group stratification			Inequality gradient		
Cancer type	Interventions	Comparators	Difference^a^	Interventions	Comparators	Difference^a^	Interventions	Comparators	Difference^a^
mBC	29.6 (26.3; 33.2)	24.2 (21.4; 27.2)	5.4 (0.9; 9.9)	71.1 (61.9; 81.5)	57.1 (49.7; 65.5)	13.9 (1.4; 26.6)	16.4 (14.4; 18.8)	13.2 (11.6; 15.1)	3.2 (0.3; 6.1)
			23% (3.6%; 44.6%)			25% (2.2%; 50.8%)			24.7% (2.4%; 49.7%)
mCRC	10.1 (9.1; 11.1)	7.3 (6.4; 8.2)	2.8 (1.4; 4.2)	23.7 (21.0; 26.7)	17.0 (14.7; 19.5)	6.7 (3.0; 10.5)	5.5 (4.9; 6.2)	3.9 (3.4; 4.5)	1.6 (0.7; 2.4)
			38.9% (18.0%; 62.3%)			40.4% (16.1%; 67.9%)			40.4% (16.1%; 67.1%)
mNSCLC	39.9 (35.9; 44.9)	30.8 (23.6; 38.1)	9.1 (0.7; 17.4)	69.3 (58.3; 89.3)	84.2 (56.1; 153.3)	−14.9 (−84.5; 19.0)	17.1 (14.7; 21.3)	19.0 (13.4; 33.1)	−2.0 (−16.1; 5.1)
			31.4% (1.8%; 71.1%)			−12.2% (−55.8%; 31.1%)			−5.5% (−49.2%; 35.1%)

^a^ Confidence intervals for the Difference that are strictly positive or negative, i.e., that do not cover zero, suggest the difference is significant.

**TABLE 3:** Survival inequalities in terms of PFS for all cancers, reported as mean (95% confidence interval) based on the probabilistic analysis.

	2 group stratification			5 group stratification			Inequality gradient		
Cancer type	Interventions	Comparators	Difference^a^	Interventions	Comparators	Difference^a^	Interventions	Comparators	Difference^a^
mBC	8.8 (8.0; 9.7)	5.5 (5.0; 6.1)	3.3 (2.3; 4.3)	21.4 (19.0; 23.9)	12.7 (11.3; 14.2)	8.7 (5.9; 11.6)	4.9 (4.4; 5.5)	3.0 (2.6; 3.3)	2.0 (1.3; 2.6)
			60.7% (39.4%; 84%)			71.7% (48.7%; 95.8%)			67.6% (42.8%; 95.2%)
mCRC	4.1 (3.7; 4.5)	0.8 (0.6; 1.1)	3.3 (2.8; 3.8)	9.1 (8.1; 10.1)	3.7 (3.1; 4.4)	5.4 (4.2; 6.6)	2.1 (1.9; 2.4)	0.8 (0.7; 0.9)	1.3 (1.1; 1.6)
			410.6% (277.4%; 563.2%)			147.6% (100.7%; 203.2%)			172.7% (121.5%; 233.3%)
mNSCLC	14.9 (13.2; 16.8)	9.2 (8.4; 10.0)	5.7 (3.8; 7.7)	37.8 (32.6; 43.6)	19.6 (17.6; 21.9)	18.2 (12.5; 24.4)	8.7 (7.5; 10.0)	4.6 (4.2; 5.1)	4.0 (2.8; 5.4)
			62.4% (39.4%; 87.7%)			93.8% (60.2%; 131.0%)			88.1% (56.7%; 122.7%)

^a^ Confidence intervals for the Difference that are strictly positive or negative, i.e., that do not cover zero, suggest the difference is significant.

### Overall survival

**FIGURE 2:**
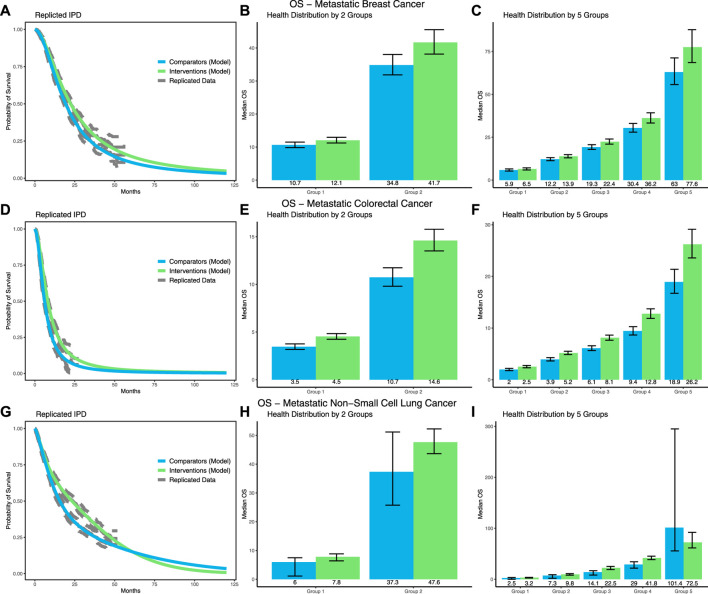
Results of the survival modeling for OS and the corresponding health distributions in terms of median OS based on the 2- and 5-group stratifications for mBC **(A–C)**, mCRC **(D–F)**, and mNSCLC **(G–I)**.

For mBC, Figure 2A illustrates the survival extrapolation using the selected log-logistic distribution, as well as the health distributions based on the 2- and 5-group stratifications. Detailed results for the survival inequalities and the differences therein are presented in Table 2. The highest increase in survival inequalities was observed for the 5-group stratification. Here, the inequality in OS increased by 13.9 (95% CI: 1.4; 26.6) months, or 25% (2.2%; 50.8%), from 57.1 (49.7; 65.5) months to 71.1 (61.9; 81.5) months.

Survival was extrapolated using a log-logistic distribution for mCRC (Figure 2D). The greatest increase in inequalities was seen in the 5-group stratification, where the inequality in OS increased by 6.7 (3.0; 10.5) months, or 40.4% (16.1%; 67.9%), from 17.0 (14.7; 19.5) months to 23.7 (21.0; 26.7) months.

For mNSCLC, survival was extrapolated using a mixture of a Gamma and a Weibull distribution (Figure 2G). The results for the 2-group stratification show an increase in OS inequality by 9.1 (0.7; 17.4) months, or 31.4% (1.8%; 71.1%), from 30.8 (23.6; 38.1) months to 39.9 (35.9; 44.9) months. Notably, however, the results for the 5-group stratification showed a nonsignificant decrease in inequalities by 14.9 (−84.5; 19.0) months, or 12.2% (−55.8%; 31.1%), from 84.2 (56.1; 153.3) months to 69.3 (58.3; 89.3) months, which is the result of the crossing of the survival curve.

### Progression-free survival

**FIGURE 3:**
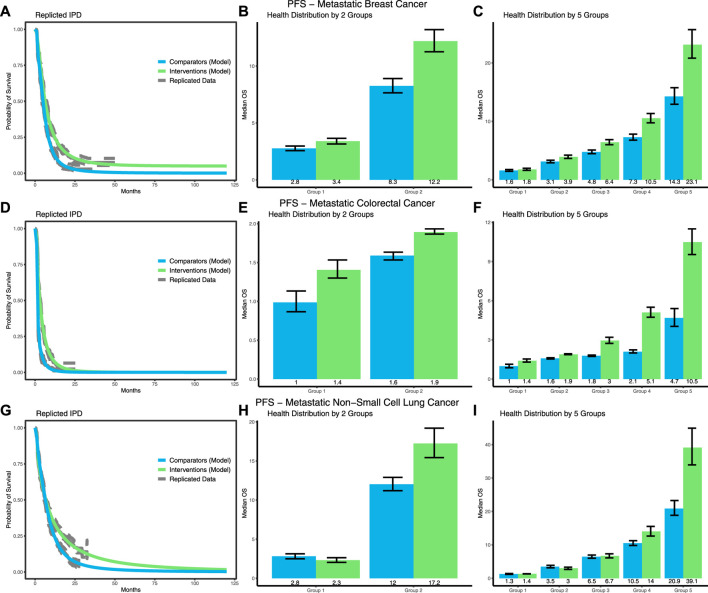
Results of the survival modeling for PFS and the corresponding health distributions in terms of median PFS based on the 2- and 5-group stratification for mBC **(A–C)**, mCRC **(D–F)**, and mNSCLC **(G–I)**.

For all cancer types, PFS was extrapolated using a mixture of a log-logistic and log-normal distribution (Figure 3A, D, G), showing significant increases in survival inequalities. For mBC (Figure 3B, C), the greatest increase in inequalities was seen in the 5-group stratification, where the inequality in PFS increased by 8.7 (5.9; 11.6) months, or 71.7% (48.7%; 95.8%), from 12.7 (11.3; 14.2) months to 21.4 (19.0; 23.9) months.

For mCRC (Figure 3E, F), the 5-group stratification again showed the greatest increase in inequalities in absolute sense. The inequality in PFS increased by 5.4 (4.2; 6.6) months, or 147.6% (100.7%; 203.2%), from 3.7 (3.1; 4.4) months to 9.1 (8.1; 10.1) months. Note that the increase was higher in relative sense for the 2-group stratification, but this was caused by the low value for the comparator group as denominator.

For mNSCLC (Figure 3H, I), the greatest increase in inequalities was seen in the 5-group stratification, where the inequality in PFS increased by 18.2 (12.5; 24.4) months, or 93.8% (60.2%; 131.0%), from 19.6 (17.6; 21.9) months to 37.8 (32.6; 43.6) months.

## Discussion

In this research, a framework was proposed to quantify the impact of novel metastatic cancer therapies on health inequalities in survival outcomes based on published KM curves. This comes at a pivotal point in time, where there is increasing debate about how to consider equity-related aspects in health economic analyses. For example, there has been a collective effort to show that lack of health equity consideration within a health technology assessment (HTA) could result in neglecting an important aspect of the value of interventions and potentially misallocation of healthcare resources (Cookson et al., 2017; Podolsky et al., 2022). Furthermore, the Institute for Clinical and Economic Review has recently published a whitepaper on the use of methods that support equity-informed analyses for HTA in the United States (Agboola et al., 2023). It has also been suggested that even the most popular method, namely, distributional cost-effectiveness analysis, faces significant challenges in implementation by HTA agencies due to scarcity and lack of consistency within equity-informed data (Meunier et al., 2023).

The framework was successfully applied to estimate the impact of novel therapies on OS and PFS outcomes in mBC, mCRC, and mNSCLC. Overall, the results of this analysis showed that the pooled novel therapies improved median survival for OS and PFS but widened survival inequalities in absolute terms by increasing survival the most among those patient groups who had comparatively better survival outcomes already. The findings for mNSCLC in terms of OS showed that the framework is also capable of identifying decreases in inequalities, albeit nonsignificant for this case study. Hypotheses on what may explain this finding are beyond the scope of this research.

Although the framework was applied to pooled therapies for certain metastatic cancers, it can be generalized to other settings as well. For instance, it can be applied to specific treatments to investigate the impact of certain therapies on inequalities. It can also be applied to other types and stages of cancer, other disease areas and treatments, and other time-to-event outcomes. The philosophy behind the framework can also be used as a foundation for exploring the quantification of health inequalities based on published distributions for other types of outcomes.

In addition to its potential broad applicability, strengths of the framework include that it is a conceptually straightforward approach to visualize and explain, and it is relatively easy to apply, with the provided R code further contributing to uptake and use by other researchers. Therefore, it represents a potentially important tool that can provide useful insights when IPD are not available, facilitating an initial understanding of how an intervention may impact healthcare disparities and informing further IPD-driven research into health disparities.

The main limitation of the proposed framework is that it does not provide any direct information as to why the changes in the health distribution occur. Although various organizations have published slightly different versions, definitions generally consider inequities or disparities as unjust differences in outcomes that can be explained by SDOH, whereas inequalities are used as a synonym for inequities or to simply describe that there are differences in outcomes (Braveman, 2014; Arcaya et al., 2015). Here, we adopt the latter definition of inequalities, and therefore, one could say that the framework provides insights into the impact on health inequalities but not disparities, which would require explanation of the changes based on SDOH. Results obtained through the framework could, hence, be complemented with disease-specific evidence on links between health inequalities and SDOH or, ideally, analyses of RCT data or real-world data to understand the impact of SDOH on the outcomes. Nevertheless, the proposed approach using aggregate data provides useful initial insights into how healthcare interventions may impact the distribution of health outcomes between groups of individuals.

A natural extension of this work would be to use the results in, for example, a distributional cost-effectiveness analysis. Further research is also warranted to apply the framework to more case studies within oncology and beyond. Finally, it would be particularly interesting to apply the framework to a case study for which the corresponding IPD are available to compare the results and to investigate the extent to which the impact on inequalities can be explained by SDOH—to assess the link between health inequalities and disparities.

## Data Availability

The original contributions presented in the study are included in the article/Supplementary Material, further inquiries can be directed to the corresponding author.
