# Supplementary material for: Quantifying the impact of novel metastatic cancer therapies on health inequalities in survival outcomes
Source: Front Pharmacol. 2023 Nov 24;14:1249998. doi: 10.3389/fphar.2023.1249998 (PMC10704132; doi:10.3389/fphar.2023.1249998)

Supplementary Material

Contents

1. The Selection Algorithm 2
2. Overall Survival (OS) 3
2.1 Metastatic Breast Cancer (mBC) 3
2.1.1 Survival Extrapolation Selection process 3
2.1.2 Feasible Extrapolations 4
2.1.3 Infeasible Extrapolations 5
2.1.4 Selected Extrapolations 6
2.2 Metastatic Colorectal Cancer (mCRC) 7
2.2.1 Survival Extrapolation Selection process 7
2.2.2 Feasible Extrapolations 8
2.2.3 Infeasible Extrapolations 9
2.2.4 Selected Extrapolations 10
2.3 Metastatic Non-Small Cell Lung Cancer (mNSCLC) 11
2.3.1 Survival Extrapolation Selection process 11
2.3.2 Feasible Extrapolations 12
2.3.3 Infeasible Extrapolations 13
2.3.4 Selected Extrapolations 14
3. Progression-Free Survival (PFS) 15
3.1 Metastatic Breast Cancer (mBC) 15
3.1.1 Survival Extrapolation Selection Process 15
3.1.2 Feasible Extrapolations 16
3.1.3 Infeasible Extrapolations 17
3.1.4 Selected Extrapolations 18
3.2 Metastatic Colorectal Cancer (mCRC) 19
3.2.1 Survival Extrapolation Selection process 19
3.2.2 Feasible Extrapolations 20
3.2.3 Infeasible Extrapolations 21
3.2.4 Selected Extrapolations 22
3.3 Metastatic Non-Small Cell Lung Cancer (mNSCLC) 23
3.3.1 Survival Extrapolation Selection process 23
3.3.2 Feasible Extrapolations 24
3.3.3 Infeasible Extrapolations 26
3.3.4 Selected Extrapolations 27

1. The Selection Algorithm

The diagram below shows a summary of the process of selecting the appropriate survival extrapolations for the survival analysis:

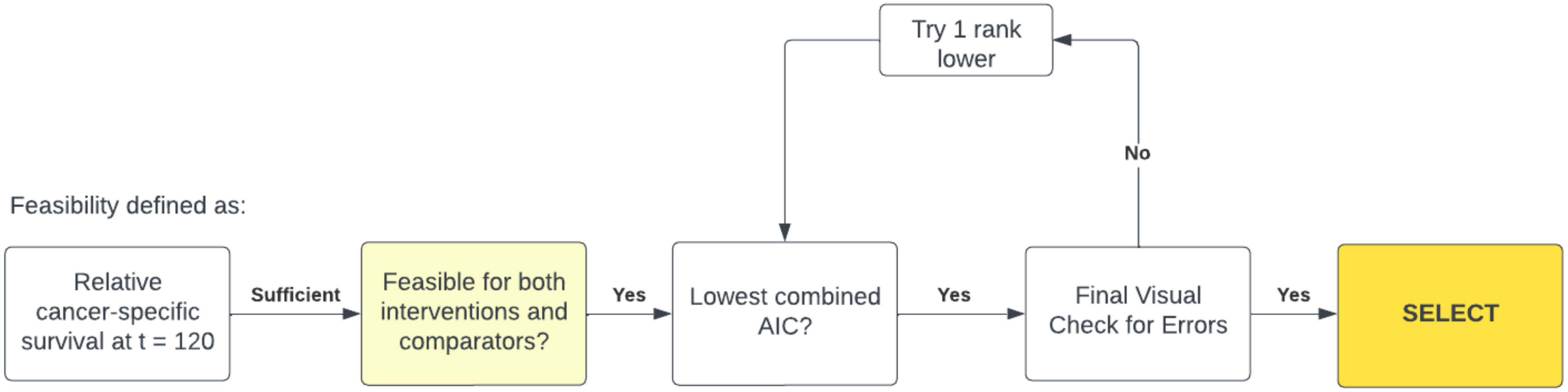

2. Overall Survival (OS)

2.1 Metastatic Breast Cancer (mBC)

2.1.1 Survival Extrapolation Selection process

		INTERVENTION					COMPARATOR					COMBINED	
	**Distribution**	**pFeasible**	**Feasible?**	**nPar**	**LogLik**	**AIC**	**pFeasible**	**Feasible?**	**nPar**	**LogLik**	**AIC**	**AIC**	**Rank**
1	gamma	0.003853	Yes	2	-2415.15	4834.291	0.001145	Yes	2	-2072.49	4148.981	8983.272	13
2	gompertz	1.08E-06	Yes	2	-2442.69	4889.378	8.96E-09	Yes	2	-2099.62	4203.236	9092.614	19
3	llogis	0.048487	Yes	2	-2410.14	4824.281	0.032366	Yes	2	-2068.36	4140.719	8965	1
4	lnorm	0.04899	Yes	2	-2415.23	4834.462	0.0279	Yes	2	-2073.55	4151.094	8985.556	14
5	weibull	0.001096	Yes	2	-2420.35	4844.698	0.000202	Yes	2	-2077.94	4159.888	9004.587	17
6	gamma / gamma	0.003853	Yes	5	-2415.15	4840.291	0.001145	Yes	5	-2072.49	4154.981	8995.272	15
7	gamma / gompertz	0.16571	Yes	5	-2409.32	4828.648	4.81E-05	Yes	5	-2068.13	4146.253	8974.901	6
8	gamma / llogis	0.066635	Yes	5	-2409.95	4829.904	0.03086	Yes	5	-2068.35	4146.705	8976.609	9
9	gamma / lnorm	0.080274	Yes	5	-2408.26	4826.512	0.029953	Yes	5	-2068.25	4146.503	8973.015	2
10	gamma / weibull	0.16019	Yes	5	-2409.33	4828.654	5.76E-05	Yes	5	-2068.1	4146.199	8974.853	5
11	gompertz / gompertz	1.09E-06	Yes	5	-2442.69	4895.378	8.97E-09	Yes	5	-2099.62	4209.236	9104.614	20
12	gompertz / llogis	0.07904	Yes	5	-2409.92	4829.837	0.031696	Yes	5	-2068.36	4146.725	8976.561	7
13	gompertz / lnorm	0.062199	Yes	5	-2410.49	4830.986	0.029792	Yes	5	-2068.57	4147.139	8978.125	12
14	gompertz / weibull	2.45E-07	Yes	5	-2409.61	4829.219	1.20E-10	Yes	5	-2068.91	4147.815	8977.034	11
15	llogis / llogis	0.048487	Yes	5	-2410.14	4830.281	0.032366	Yes	5	-2068.36	4146.719	8977	10
16	llogis / lnorm	0.071748	Yes	5	-2408.37	4826.73	0.029729	Yes	5	-2068.34	4146.68	8973.411	3
17	llogis / weibull	0.078993	Yes	5	-2409.92	4829.834	0.029293	Yes	5	-2068.37	4146.739	8976.573	8
18	lnorm / lnorm	0.04899	Yes	5	-2415.23	4840.462	0.0279	Yes	5	-2073.55	4157.094	8997.556	16
19	lnorm / weibull	0.08942	Yes	5	-2408.55	4827.102	0.036147	Yes	5	-2068.31	4146.623	8973.725	4
20	weibull / weibull	0.001096	Yes	5	-2420.35	4850.698	0.000202	Yes	5	-2077.94	4165.888	9016.587	18

Table 1:Survival Extrapolation Selection Process for Overall Survival (OS) - Metastatic Breast Cancer (mBC).

Note: the light-yellow row show all feasible extrapolations, and the dark yellow row shows the selected extrapolation.

2.1.2 Feasible Extrapolations

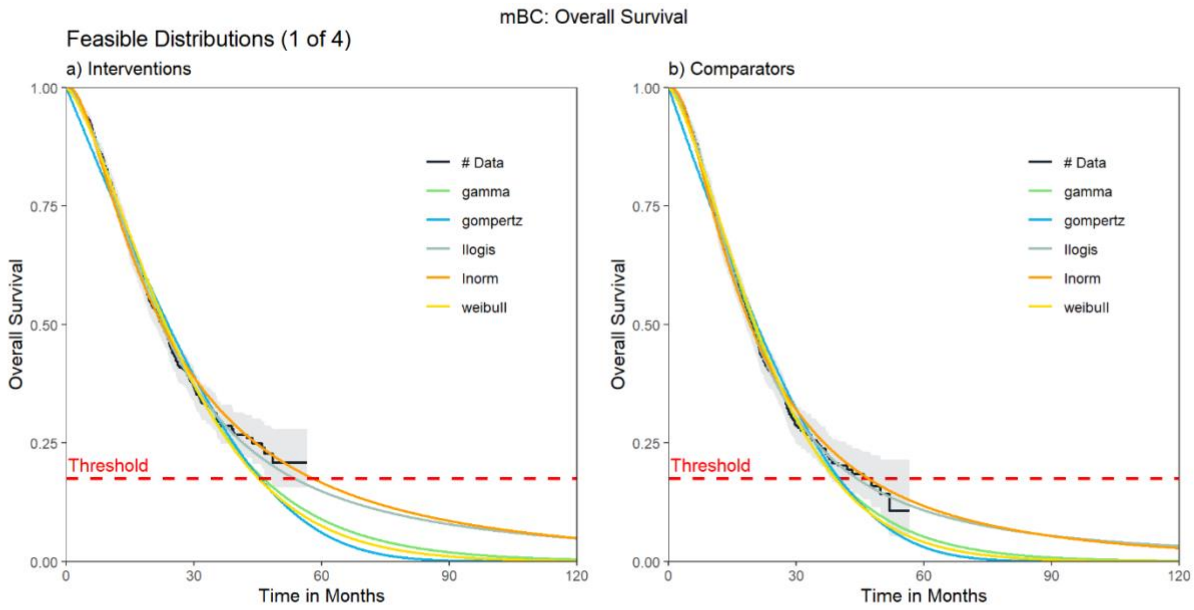

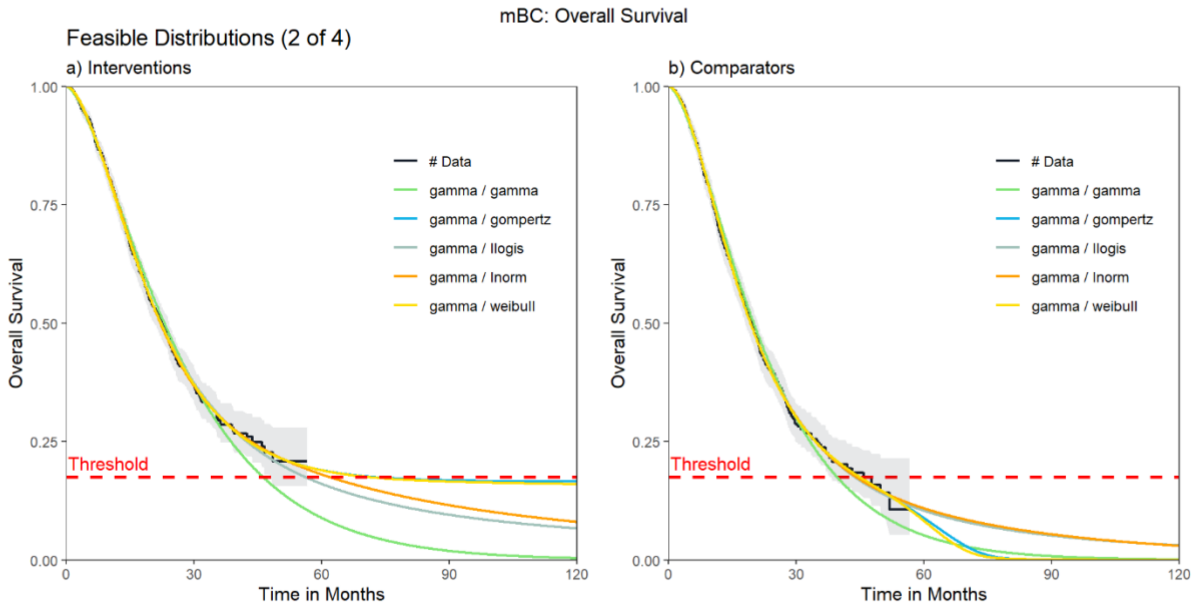

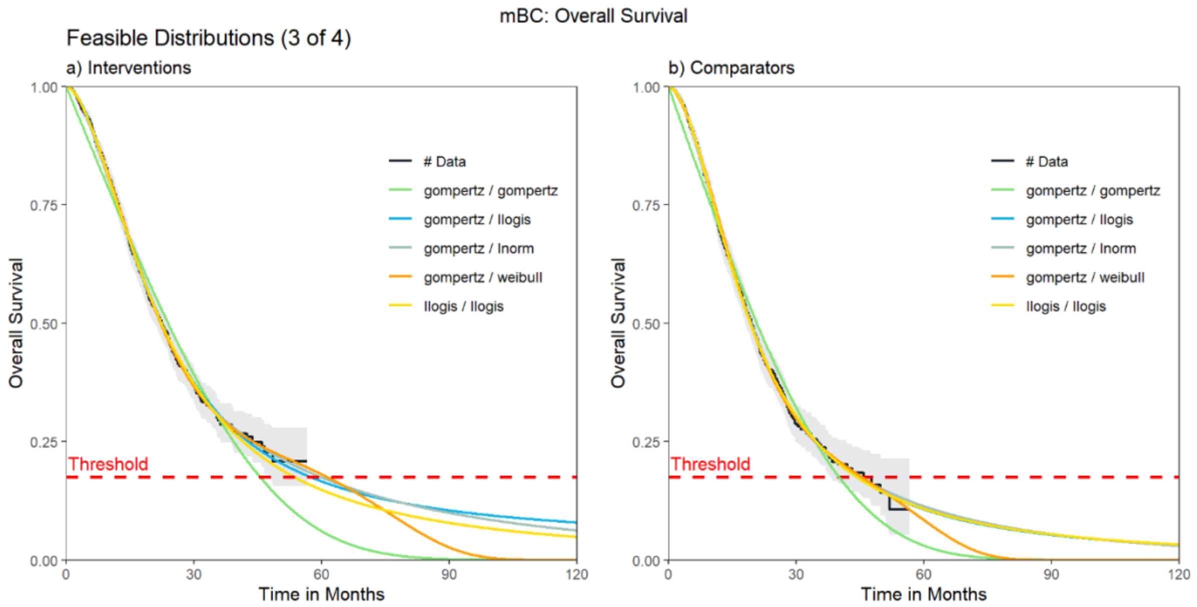

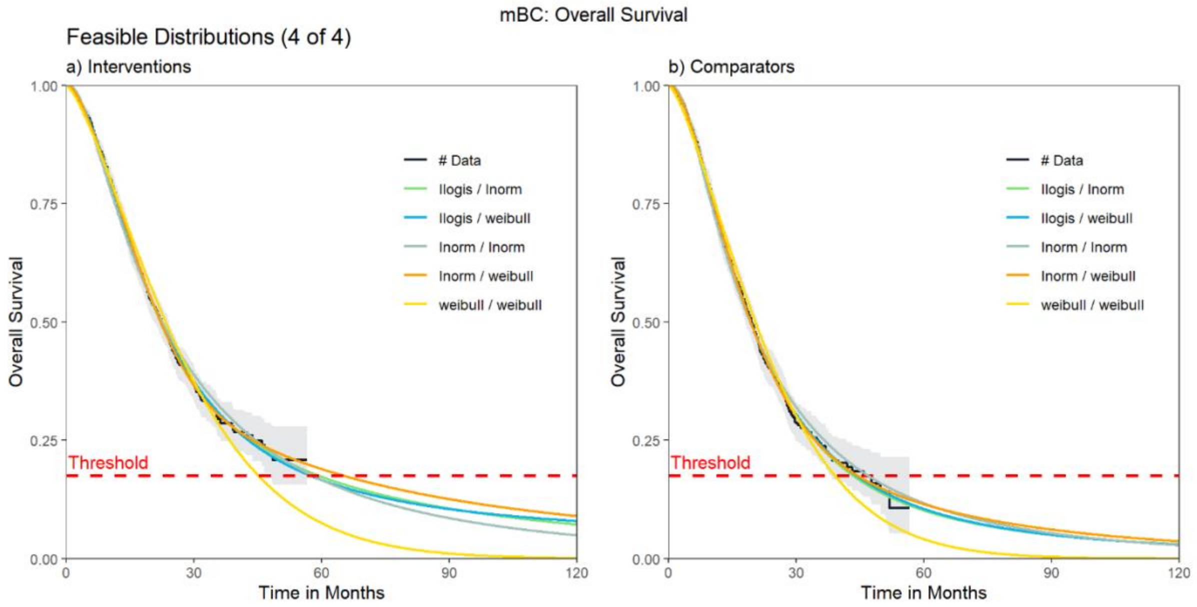

2.1.3 Infeasible Extrapolations

There were no infeasible distributions for mBC – OS.

2.1.4 Selected Extrapolations

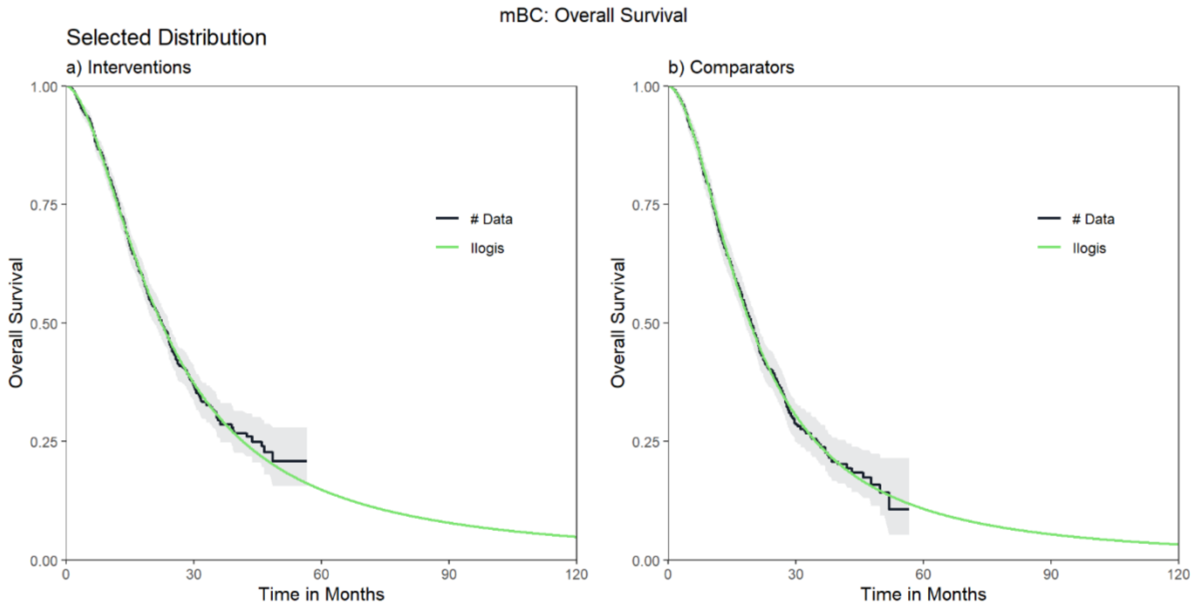

2.2 Metastatic Colorectal Cancer (mCRC)

2.2.1 Survival Extrapolation Selection process

		INTERVENTION					COMPARATOR					COMBINED	
	**Distribution**	**pFeasible**	**Feasible?**	**nPar**	**LogLik**	**AIC**	**pFeasible**	**Feasible?**	**nPar**	**LogLik**	**AIC**	**AIC**	**Rank**
1	gamma	2.16E-08	Yes	2	-2136.12	4276.242	2.26E-11	Yes	2	-1334.77	2673.54	6949.782	13
2	gompertz	0	Yes	2	-2175.6	4355.204	0	Yes	2	-1360.74	2725.487	7080.691	19
3	llogis	0.006307	Yes	2	-2127.53	4259.061	0.003045	Yes	2	-1331.08	2666.158	6925.219	1
4	lnorm	0.002372	Yes	2	-2137.31	4278.613	0.000483	Yes	2	-1334.11	2672.219	6950.832	14
5	weibull	1.81E-12	Yes	2	-2144.79	4293.571	0	Yes	2	-1340.97	2685.93	6979.501	17
6	gamma / gamma	2.16E-08	Yes	5	-2136.12	4282.242	2.25E-11	Yes	5	-1334.77	2679.54	6961.782	15
7	gamma / gompertz	5.29E-14	Yes	5	-2127.47	4264.933	0	Yes	5	-1327.66	2665.326	6930.259	10
8	gamma / llogis	0.005756	Yes	5	-2127.4	4264.799	0.000515	Yes	5	-1327.58	2665.166	6929.966	7
9	gamma / lnorm	0.001772	Yes	5	-2127.59	4265.184	7.58E-10	Yes	5	-1326.8	2663.595	6928.779	5
10	gamma / weibull	7.65E-05	Yes	5	-2127.37	4264.74	8.08E-17	Yes	5	-1326.67	2663.342	6928.082	3
11	gompertz / gompertz	0	Yes	5	-2175.6	4361.204	0	Yes	5	-1360.74	2731.487	7092.691	20
12	gompertz / llogis	0.021473	Yes	5	-2127.38	4264.766	0.000228	Yes	5	-1327.19	2664.382	6929.147	6
13	gompertz / lnorm	0.016916	Yes	5	-2127.88	4265.751	1.00E-06	Yes	5	-1327.24	2664.488	6930.239	9
14	gompertz / weibull	0	Yes	5	-2129.5	4268.997	0	Yes	5	-1330.23	2670.456	6939.453	12
15	llogis / llogis	0.006307	Yes	5	-2127.53	4265.061	0.003045	Yes	5	-1331.08	2672.158	6937.219	11
16	llogis / lnorm	0.006935	Yes	5	-2127.45	4264.893	0.000569	Yes	5	-1327.62	2665.233	6930.126	8
17	llogis / weibull	0.005776	Yes	5	-2127.4	4264.798	5.10E-06	Yes	5	-1326.54	2663.082	6927.88	2
18	lnorm / lnorm	0.002372	Yes	5	-2137.31	4284.613	0.000483	Yes	5	-1334.11	2678.219	6962.832	16
19	lnorm / weibull	0.00232	Yes	5	-2127.21	4264.417	1.58E-11	Yes	5	-1327.1	2664.198	6928.615	4
20	weibull / weibull	1.81E-12	Yes	5	-2144.79	4299.571	0	Yes	5	-1340.97	2691.93	6991.501	18

Table 2: Survival Extrapolation Selection Process for Overall Survival (OS) - Metastatic Colorectal Cancer (mCRC)

2.2.2 Feasible Extrapolations

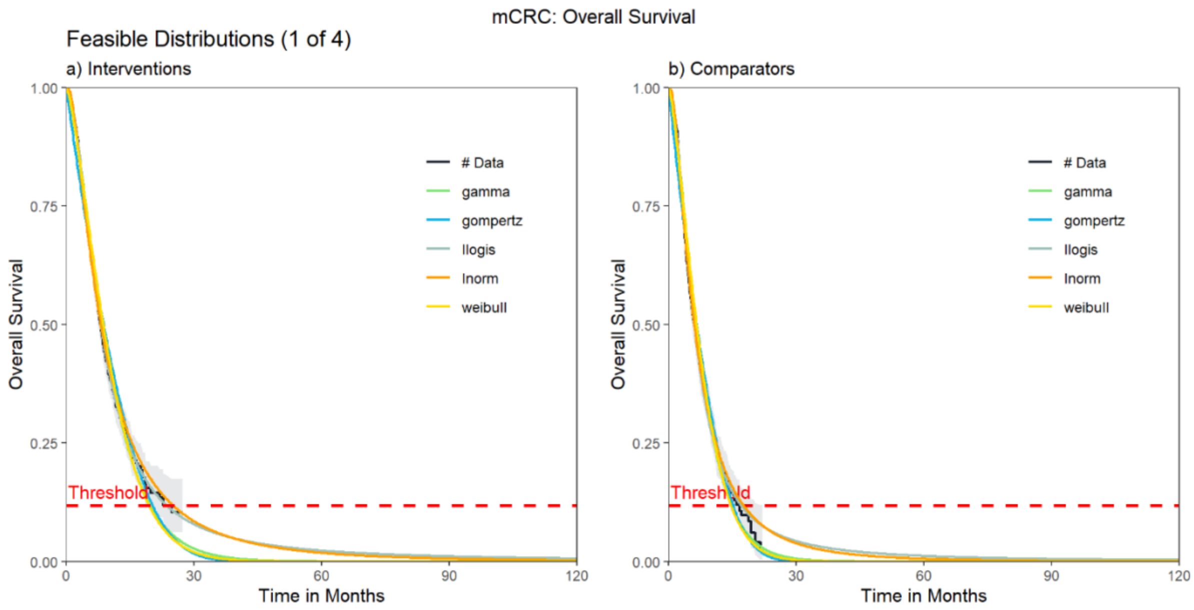

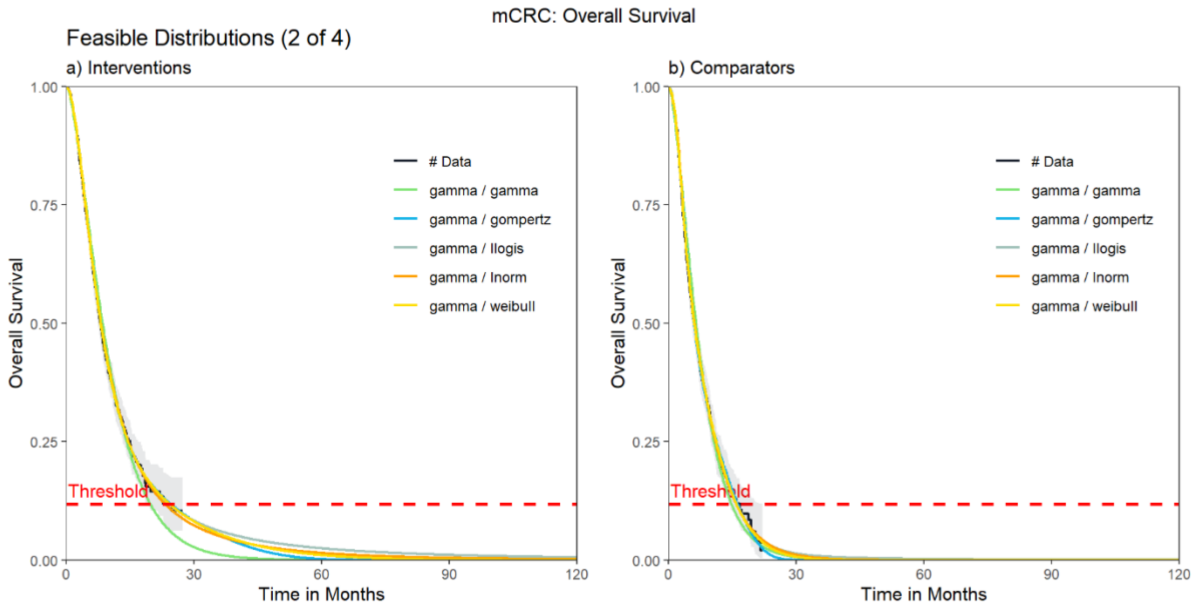

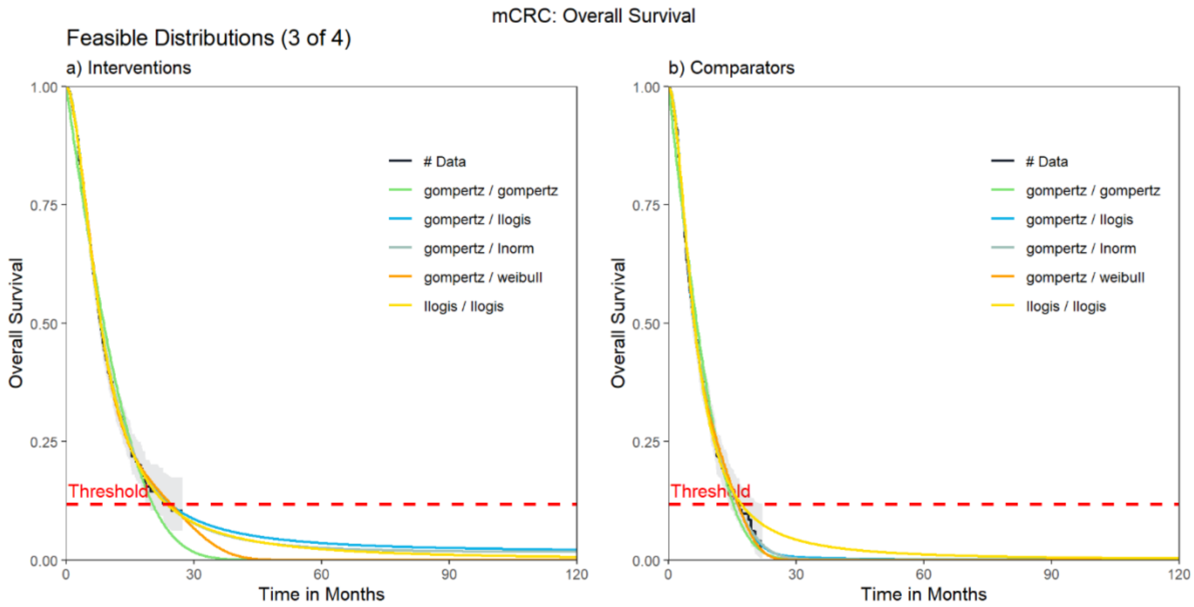

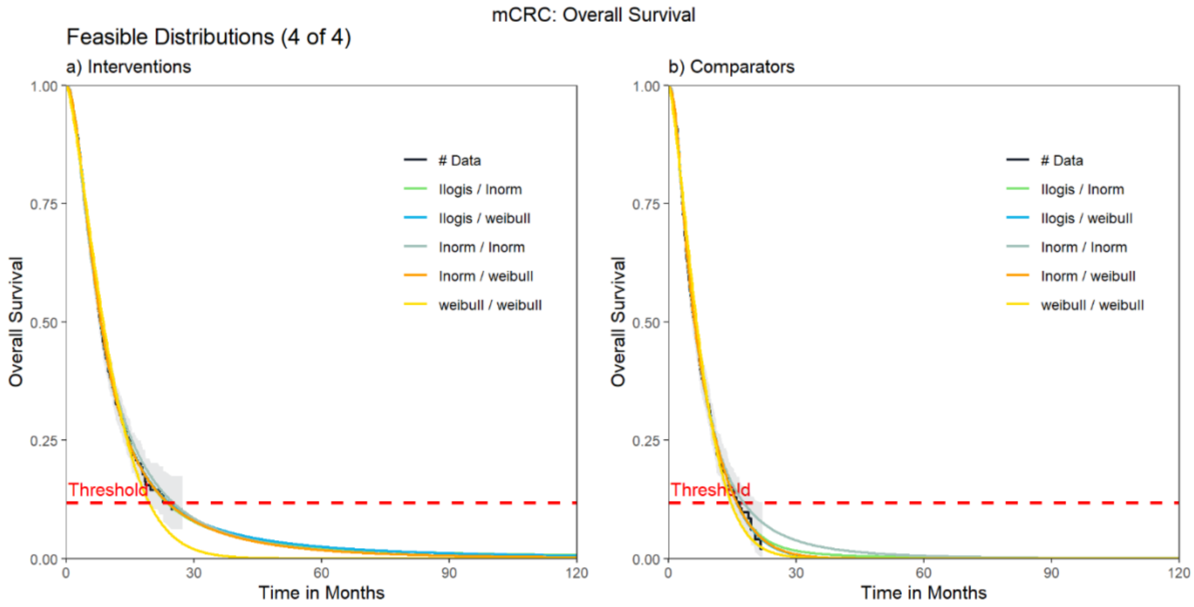

2.2.3 Infeasible Extrapolations

There were no infeasible distributions for mCRC – OS.

2.2.4 Selected Extrapolations

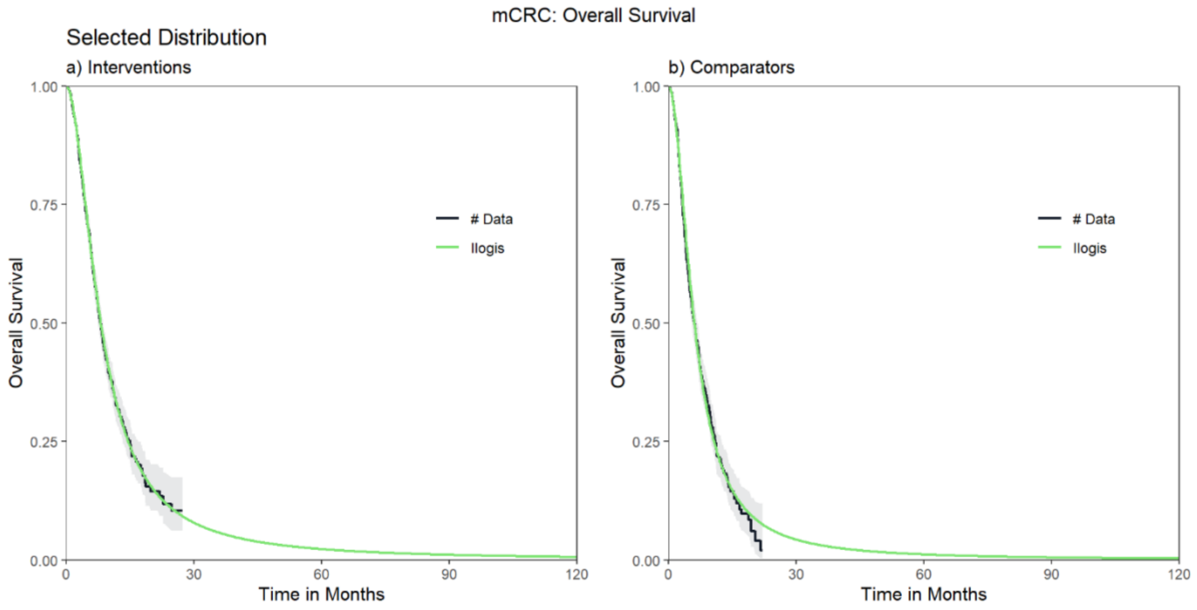

2.3 Metastatic Non-Small Cell Lung Cancer (mNSCLC)

2.3.1 Survival Extrapolation Selection process

		INTERVENTION					COMPARATOR					COMBINED	
	**Distribution**	**pFeasible**	**Feasible?**	**nPar**	**LogLik**	**AIC**	**pFeasible**	**Feasible?**	**nPar**	**LogLik**	**AIC**	**AIC**	**Rank**
1	gamma	0.027801	Yes	2	-3116.34	6236.683	0.007191	Yes	2	-2984.19	5972.386	12209.07	6
2	gompertz	0.07241	Yes	2	-3113.67	6231.339	0.052696	Yes	2	-2978.62	5961.25	12192.59	4
3	llogis	0.113155	No	NA	NA	NA	0.062147	Yes	2	-2963.79	5931.575	NA	NA
4	lnorm	0.11946	No	NA	NA	NA	0.060733	Yes	2	-2966.41	5936.818	NA	NA
5	weibull	0.031539	Yes	2	-3115.89	6235.777	0.008635	Yes	2	-2984.74	5973.489	12209.27	7
6	gamma / gamma	0.027811	Yes	5	-3116.34	6242.683	0.007191	Yes	5	-2984.19	5978.386	12221.07	8
7	gamma / gompertz	0.029995	Yes	5	-3103.98	6217.959	0.000617	Yes	5	-2962.73	5935.467	12153.43	2
8	gamma / llogis	0.020729	Yes	5	-3100.28	6210.563	0.087162	No	NA	NA	NA	NA	NA
9	gamma / lnorm	0.098599	No	NA	NA	NA	0.075922	No	NA	NA	NA	NA	NA
10	gamma / weibull	0.005808	Yes	5	-3100.05	6210.091	0.016115	Yes	5	-2962.6	5935.195	12145.29	1
11	gompertz / gompertz	0.07241	Yes	5	-3113.67	6237.339	0.052763	Yes	5	-2978.62	5967.25	12204.59	5
12	gompertz / llogis	0.009603	Yes	5	-3101.18	6212.351	0.093621	No	NA	NA	NA	NA	NA
13	gompertz / lnorm	0.104172	No	NA	NA	NA	0.091259	No	NA	NA	NA	NA	NA
14	gompertz / weibull	0.022248	Yes	5	-3104.17	6218.333	0.00126	Yes	5	-2962.99	5935.987	12154.32	3
15	llogis / llogis	0.113147	No	NA	NA	NA	0.062141	Yes	5	-2963.79	5937.575	NA	NA
16	llogis / lnorm	0.095411	No	NA	NA	NA	0.076683	No	NA	NA	NA	NA	NA
17	llogis / weibull	0.013204	Yes	5	-3100.46	6210.926	0.085601	No	NA	NA	NA	NA	NA
18	lnorm / lnorm	0.11946	No	NA	NA	NA	0.060733	Yes	5	-2966.41	5942.818	NA	NA
19	lnorm / weibull	0.102538	No	NA	NA	NA	0.076492	No	NA	NA	NA	NA	NA
20	weibull / weibull	0.031539	Yes	5	-3115.89	6241.777	0.008635	Yes	5	-2984.74	5979.489	12221.27	9

Table 3: Survival Extrapolation Selection Process for Overall Survival (OS) - Metastatic Non-Small Cell Lung Cancer (mNSCLC)

2.3.2 Feasible Extrapolations

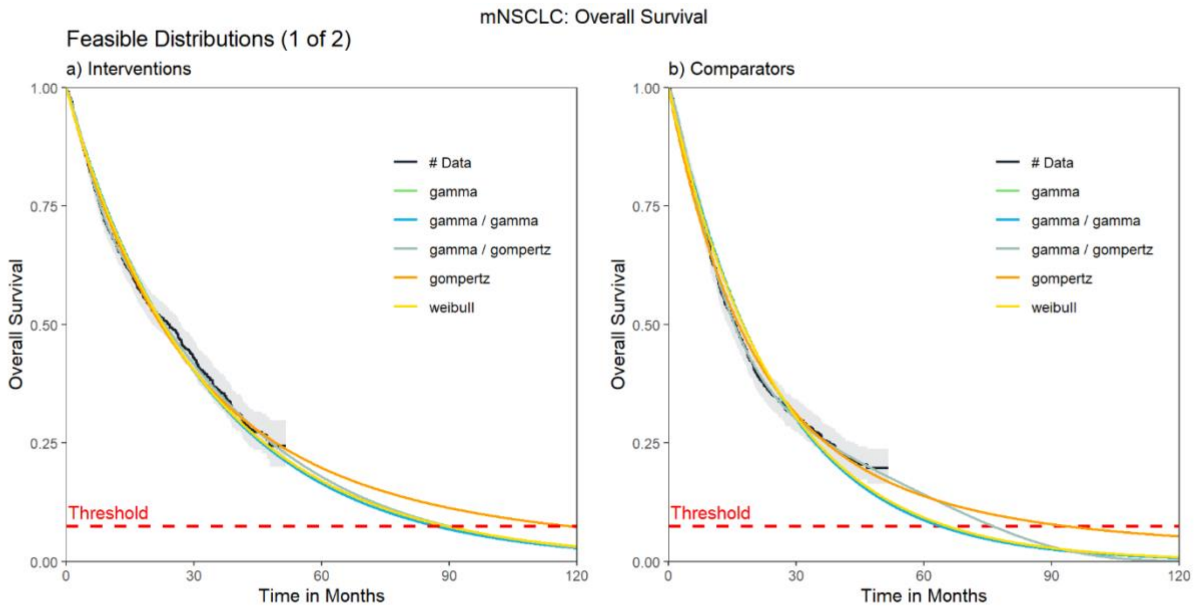

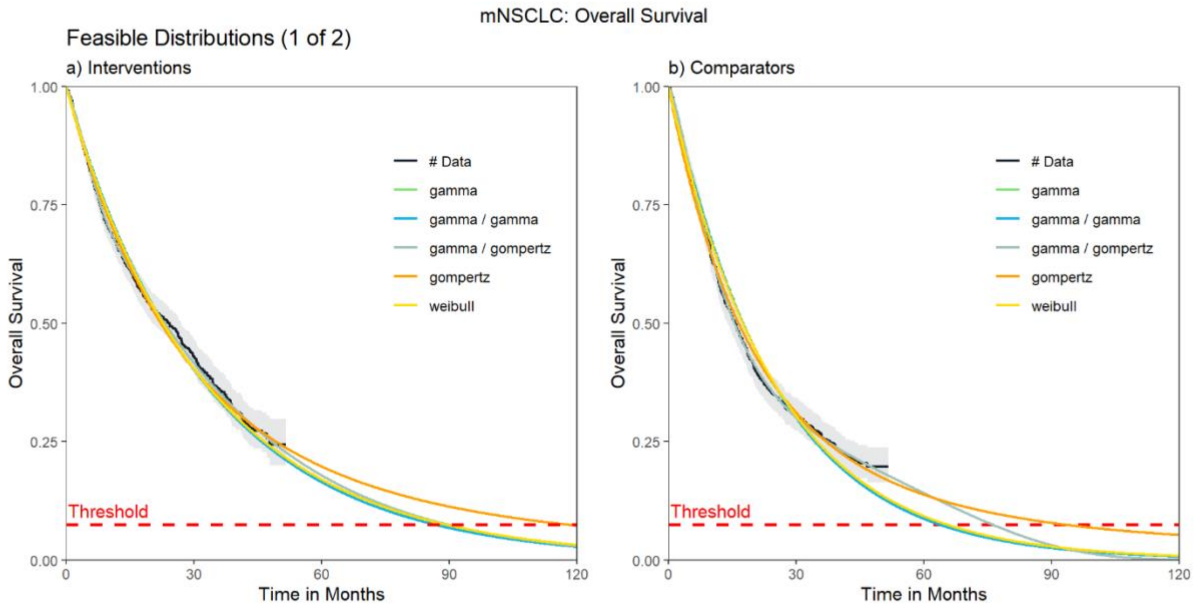

2.3.3 Infeasible Extrapolations

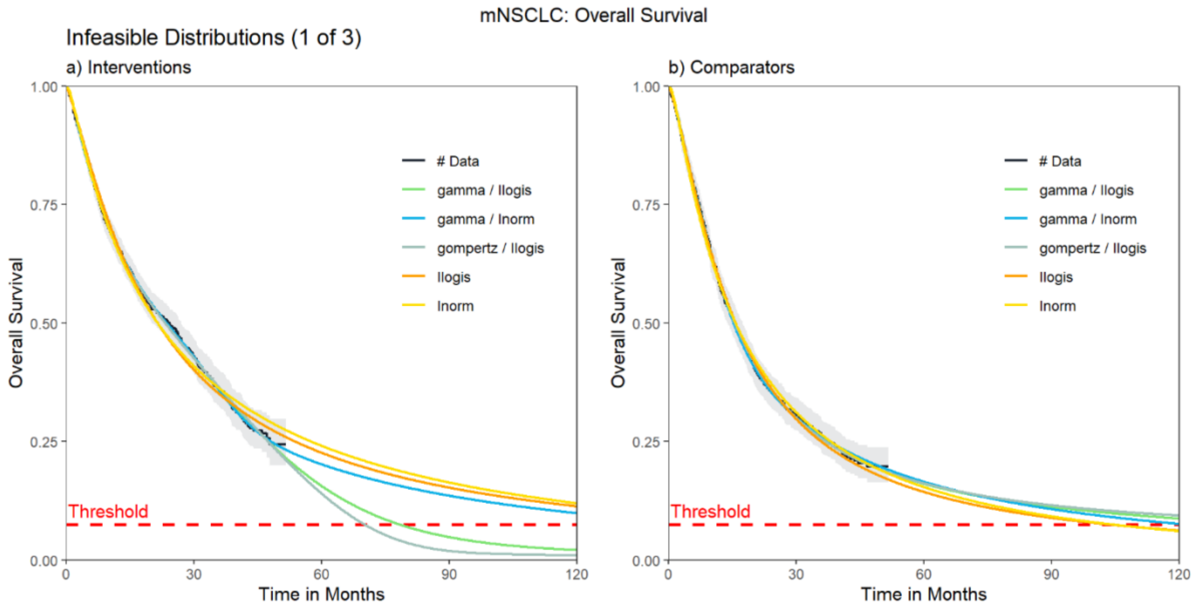

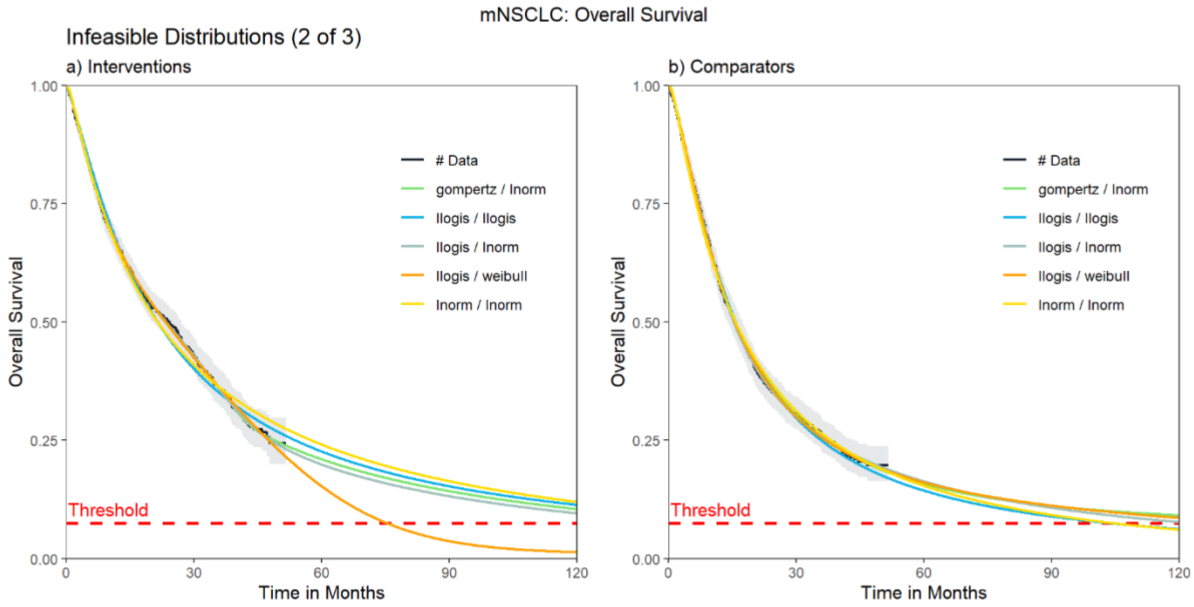

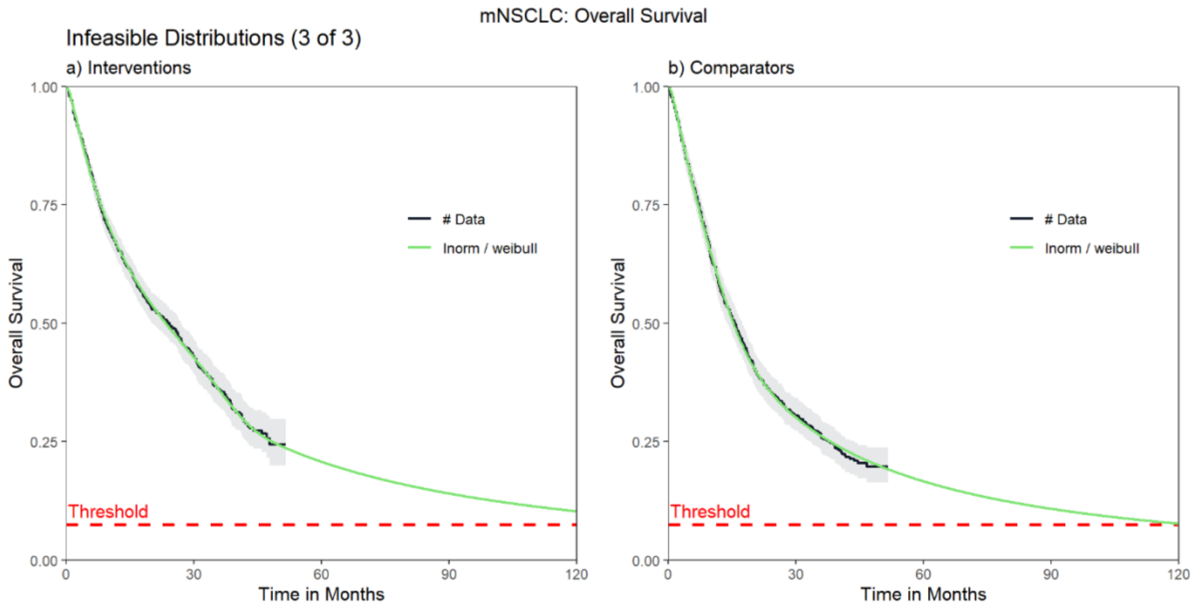

2.3.4 Selected Extrapolations

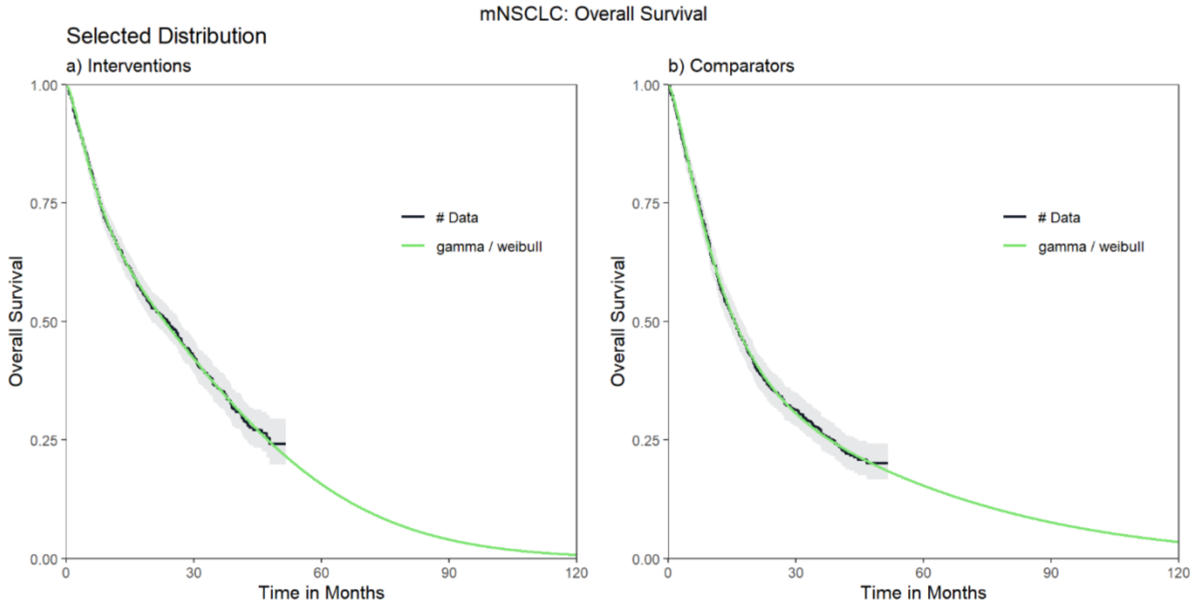

3. Progression-Free Survival (PFS)

3.1 Metastatic Breast Cancer (mBC)

3.1.1 Survival Extrapolation Selection Process

		INTERVENTION					COMPARATOR					COMBINED	
	**Distribution**	**pFeasible**	**Feasible?**	**nPar**	**LogLik**	**AIC**	**pFeasible**	**Feasible?**	**nPar**	**LogLik**	**AIC**	**AIC**	**Rank**
1	gamma	2.28E-07	Yes	2	-2420.85	4845.692	2.53E-13	Yes	2	-1729.57	3463.131	8308.823	15
2	gompertz	0.001643	Yes	2	-2435.89	4875.784	0	Yes	2	-1775.38	3554.759	8430.543	19
3	llogis	0.006506	Yes	2	-2373.74	4751.477	0.001536	Yes	2	-1711.16	3426.314	8177.79	10
4	lnorm	0.00172	Yes	2	-2365.79	4735.588	7.49E-05	Yes	2	-1701.8	3407.592	8143.181	6
5	weibull	1.74E-07	Yes	2	-2431.63	4867.27	0	Yes	2	-1745.11	3494.214	8361.484	17
6	gamma / gamma	2.28E-07	Yes	5	-2420.85	4851.692	2.54E-13	Yes	5	-1729.57	3469.131	8320.823	16
7	gamma / gompertz	0.001294	Yes	5	-2373.33	4756.66	0.000108	Yes	5	-1711.8	3433.593	8190.253	13
8	gamma / llogis	0.004239	Yes	5	-2339.05	4688.105	0.000909	Yes	5	-1707.77	3425.536	8113.64	4
9	gamma / lnorm	0.00242	Yes	5	-2365.52	4741.038	5.89E-05	Yes	5	-1700.92	3411.832	8152.87	7
10	gamma / weibull	0.001665	Yes	5	-2372.4	4754.803	0.000207	Yes	5	-1711.55	3433.096	8187.899	11
11	gompertz / gompertz	0.001643	Yes	5	-2435.89	4881.784	0	Yes	5	-1775.38	3560.759	8442.543	20
12	gompertz / llogis	0.042291	Yes	5	-2370.11	4750.228	0.001156	Yes	5	-1698.39	3406.79	8157.018	9
13	gompertz / lnorm	0.049695	Yes	5	-2359.62	4729.242	4.47E-05	Yes	5	-1691	3392	8121.242	5
14	gompertz / weibull	0.001023	Yes	5	-2382.23	4774.462	1.36E-06	Yes	5	-1721.54	3453.078	8227.54	14
15	llogis / llogis	0.006501	Yes	5	-2373.74	4757.476	0.001536	Yes	5	-1711.16	3432.314	8189.79	12
16	llogis / lnorm	0.049886	Yes	5	-2359.62	4729.234	0.000666	Yes	5	-1648.7	3307.404	8036.638	1
17	llogis / weibull	0.004325	Yes	5	-2324.86	4659.722	0.001157	Yes	5	-1698.37	3406.744	8066.467	2
18	lnorm / lnorm	0.00172	Yes	5	-2365.79	4741.588	7.49E-05	Yes	5	-1701.8	3413.592	8155.181	8
19	lnorm / weibull	0.001937	Yes	5	-2350.03	4710.063	4.49E-05	Yes	5	-1690.97	3391.949	8102.012	3
20	weibull / weibull	1.74E-07	Yes	5	-2431.63	4873.27	0	Yes	5	-1745.11	3500.214	8373.484	18

Table 4: Survival Extrapolation Selection Process for Progression-Free Survival (PFS) - Metastatic Breast Cancer (mBC)

3.1.2 Feasible Extrapolations

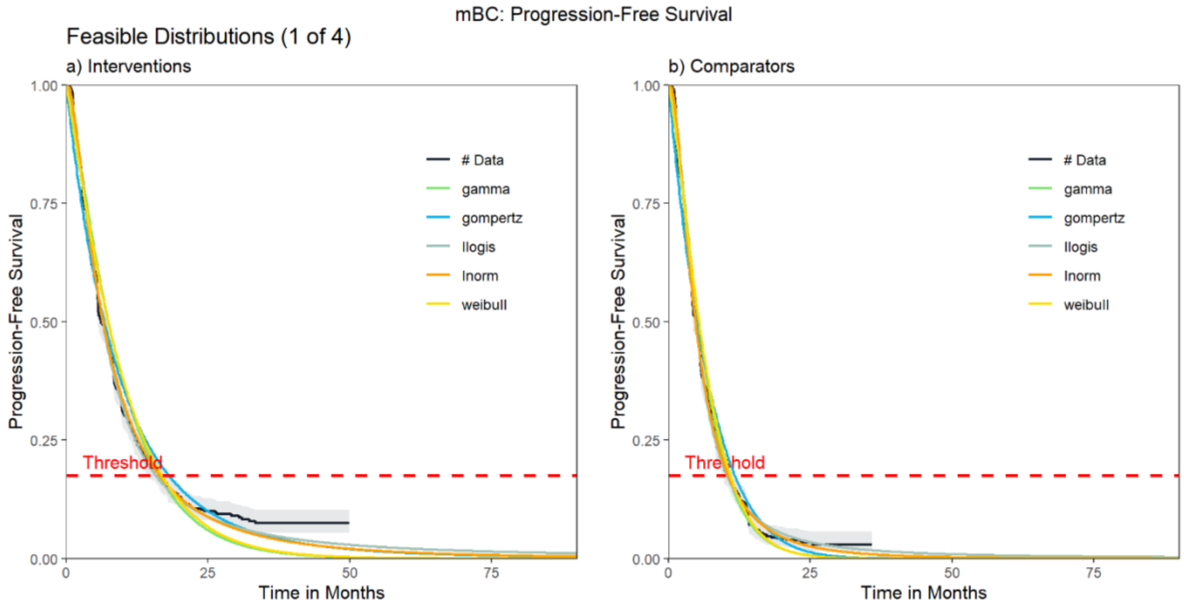

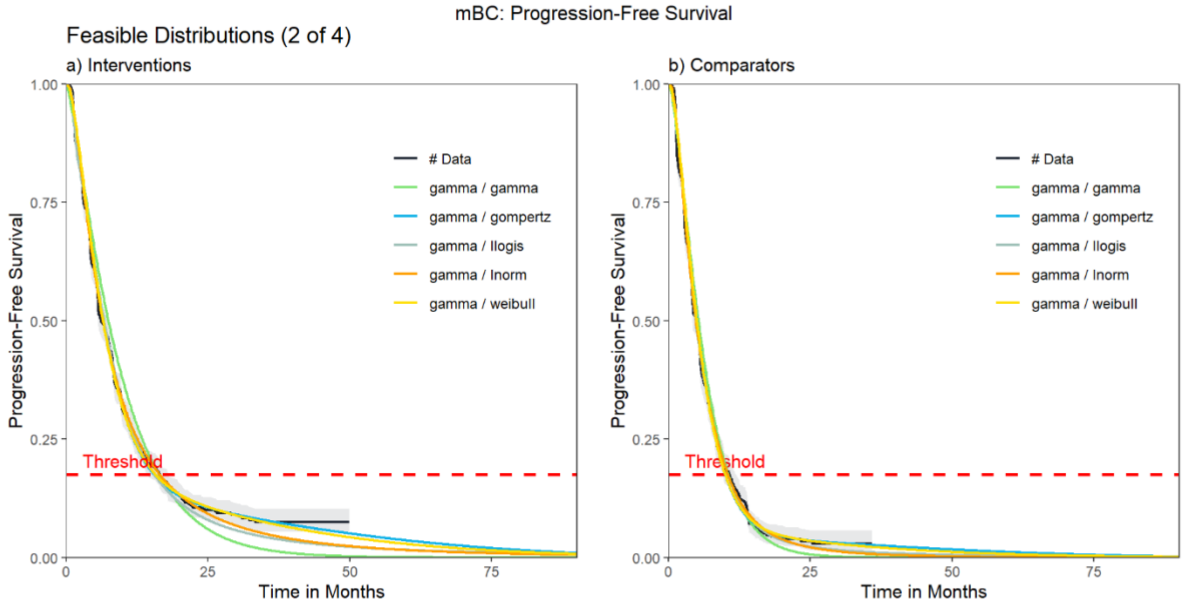

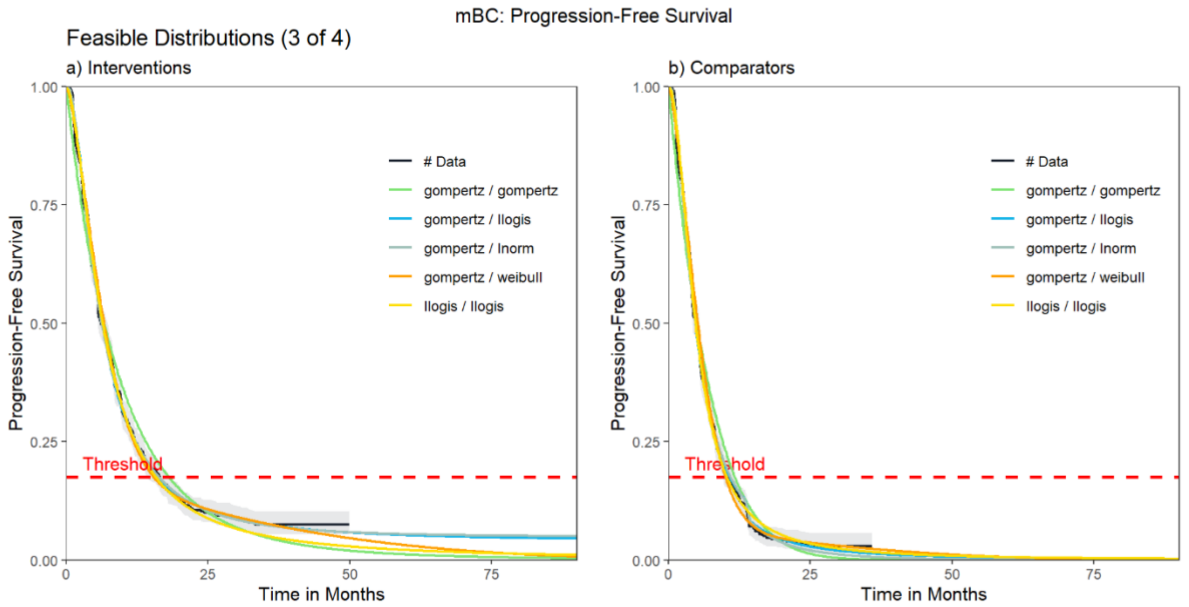

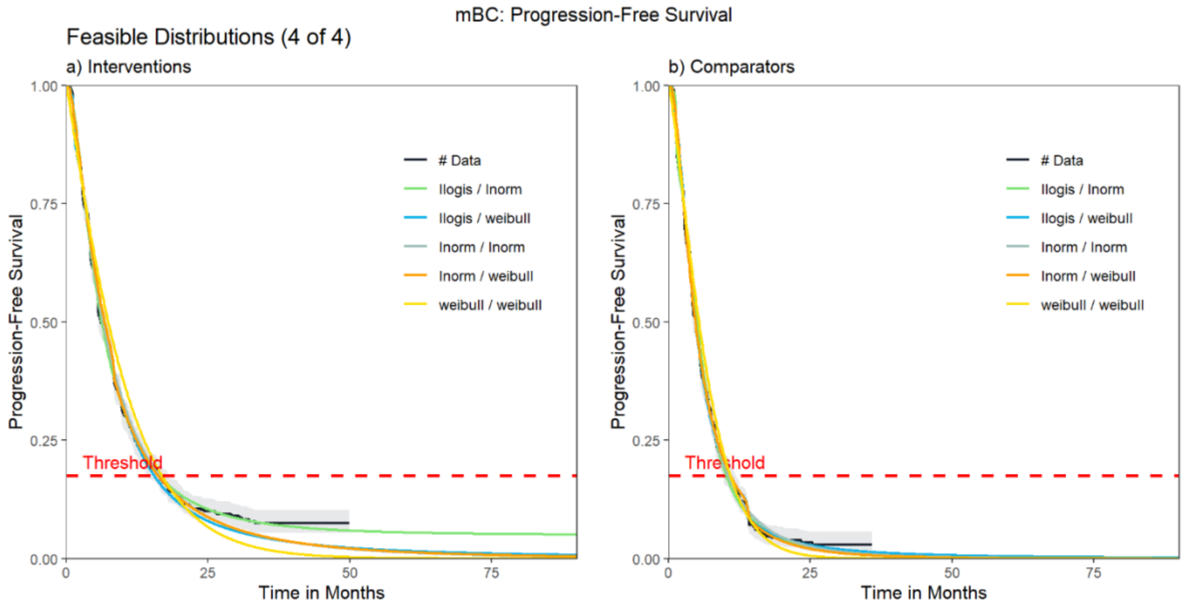

3.1.3 Infeasible Extrapolations

There were no infeasible extrapolations for metastatic breast cancer.

3.1.4 Selected Extrapolations

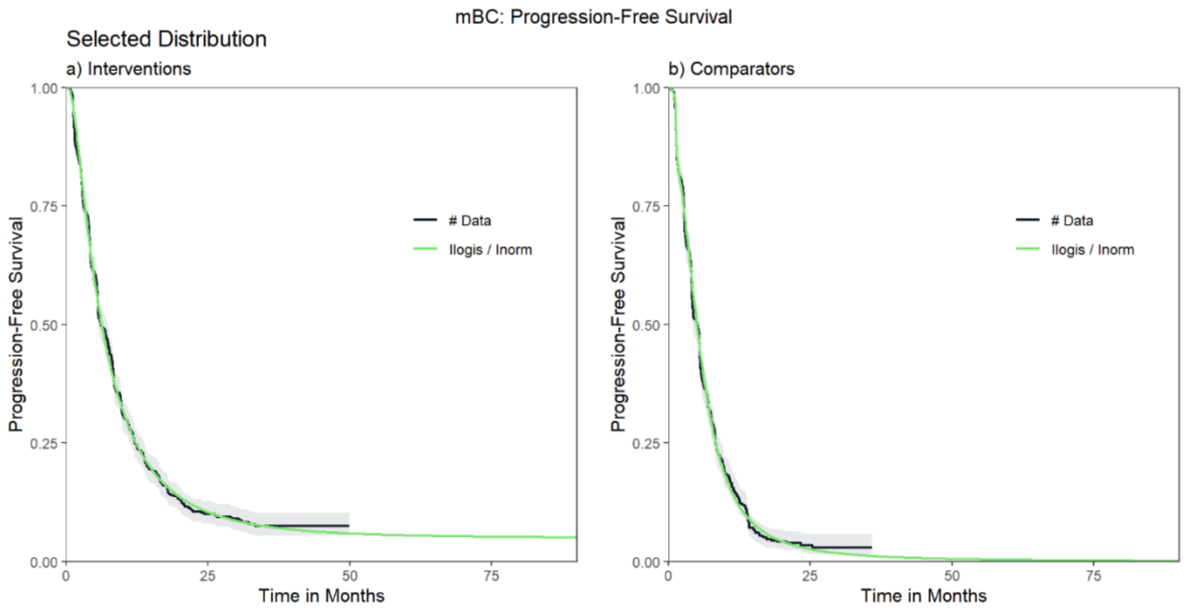

3.2 Metastatic Colorectal Cancer (mCRC)

3.2.1 Survival Extrapolation Selection process

		INTERVENTION					COMPARATOR					COMBINED	
	**Distribution**	**pFeasible**	**Feasible?**	**nPar**	**LogLik**	**AIC**	**pFeasible**	**Feasible?**	**nPar**	**LogLik**	**AIC**	**AIC**	**Rank**
1	gamma	0	Yes	2	-1945	3894.008	0	Yes	2	-862.825	1729.651	5623.659	15
2	gompertz	0	Yes	2	-2016.2	4036.409	0	Yes	2	-970.489	1944.977	5981.386	19
3	llogis	0.000453	Yes	2	-1901.34	3806.676	1.52E-06	Yes	2	-776.684	1557.368	5364.043	11
4	lnorm	4.97E-06	Yes	2	-1900.07	3804.137	1.94E-11	Yes	2	-821.545	1647.09	5451.227	13
5	weibull	0	Yes	2	-1971.2	3946.409	0	Yes	2	-909.169	1822.337	5768.746	17
6	gamma / gamma	0	Yes	5	-1945	3900.008	0	Yes	5	-862.825	1735.651	5635.659	16
7	gamma / gompertz	0	Yes	5	-1901.27	3812.534	9.94E-11	Yes	5	-704.785	1419.57	5232.103	9
8	gamma / llogis	0	Yes	5	-1794.3	3598.608	0	Yes	5	-694.935	1399.871	4998.478	4
9	gamma / lnorm	0	Yes	5	-1793.16	3596.319	1.45E-06	Yes	5	-682.203	1374.407	4970.726	2
10	gamma / weibull	0	Yes	5	-1816.56	3643.126	0	Yes	5	-700.218	1410.435	5053.561	6
11	gompertz / gompertz	0	Yes	5	-2016.2	4042.409	0	Yes	5	-970.489	1950.977	5993.386	20
12	gompertz / llogis	0	Yes	5	-1837.76	3685.512	1.62E-08	Yes	5	-701.514	1413.028	5098.54	7
13	gompertz / lnorm	7.92E-11	Yes	5	-1885.87	3781.746	1.49E-09	Yes	5	-708.112	1426.224	5207.97	8
14	gompertz / weibull	0	Yes	5	-1920.35	3850.706	0	Yes	5	-689.442	1388.883	5239.589	10
15	llogis / llogis	0.000451	Yes	5	-1901.34	3812.675	1.52E-06	Yes	5	-776.684	1563.368	5376.043	12
16	llogis / lnorm	3.04E-05	Yes	5	-1791.14	3592.275	1.94E-06	Yes	5	-682.67	1375.339	4967.614	1
17	llogis / weibull	0	Yes	5	-1805.08	3620.151	0	Yes	5	-698.454	1406.907	5027.058	5
18	lnorm / lnorm	4.97E-06	Yes	5	-1900.07	3810.137	1.94E-11	Yes	5	-821.545	1653.09	5463.227	14
19	lnorm / weibull	0	Yes	5	-1804.34	3618.68	2.40E-06	Yes	5	-671.997	1353.994	4972.674	3
20	weibull / weibull	0	Yes	5	-1971.2	3952.409	0	Yes	5	-909.169	1828.337	5780.746	18

Table 5: Survival Extrapolation Selection Process for Progression-Free Survival (PFS) - Metastatic Colorectal Cancer (mCRC)

3.2.2 Feasible Extrapolations

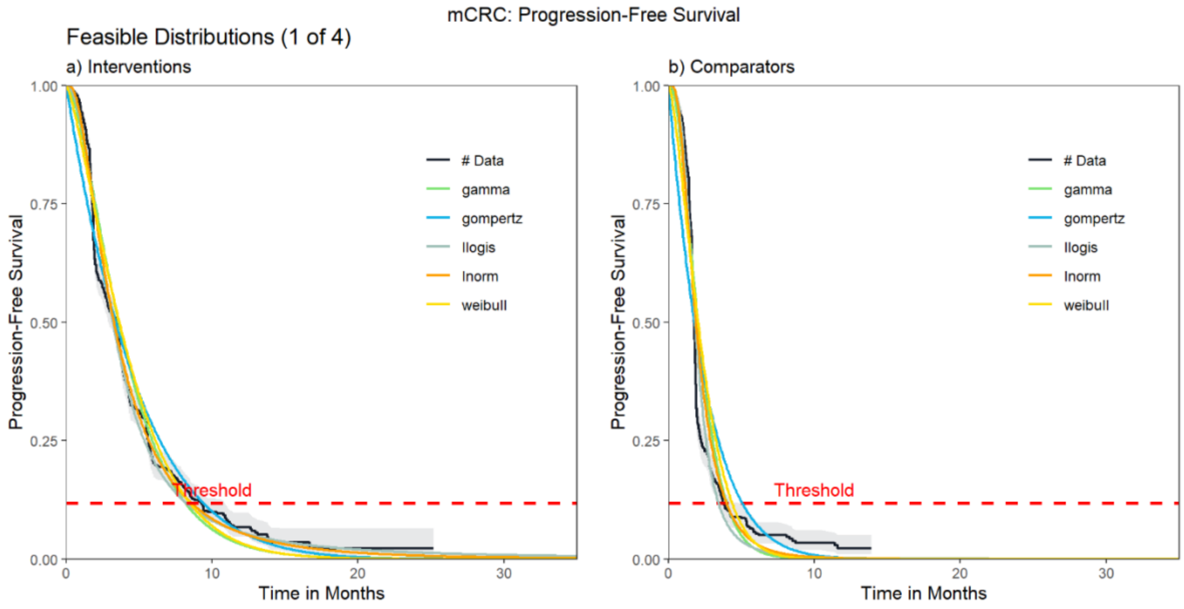

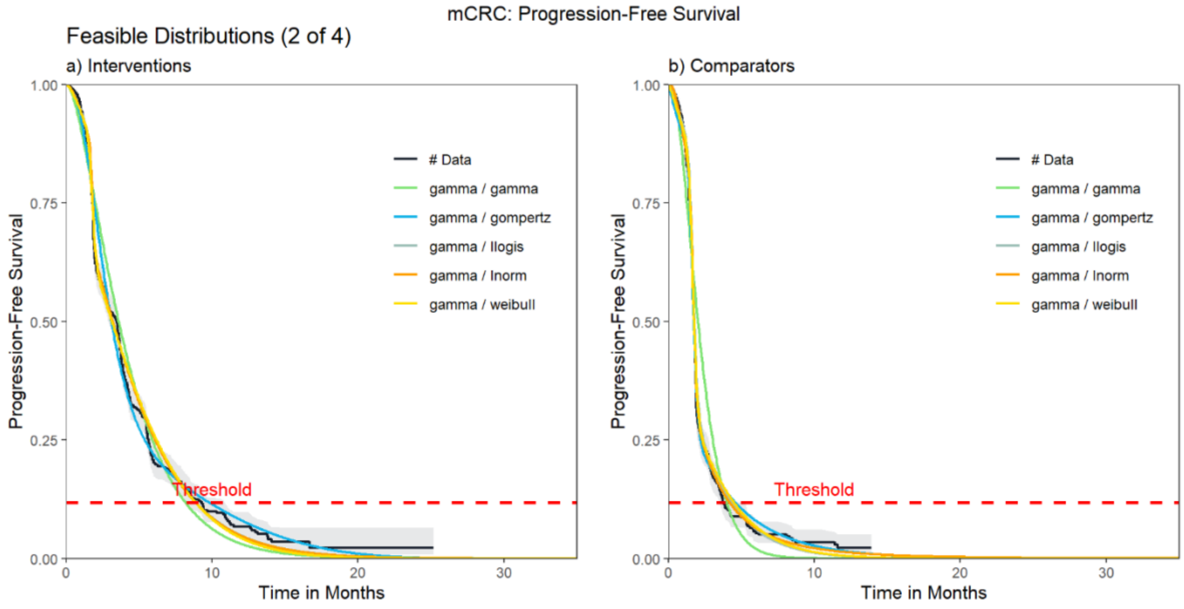

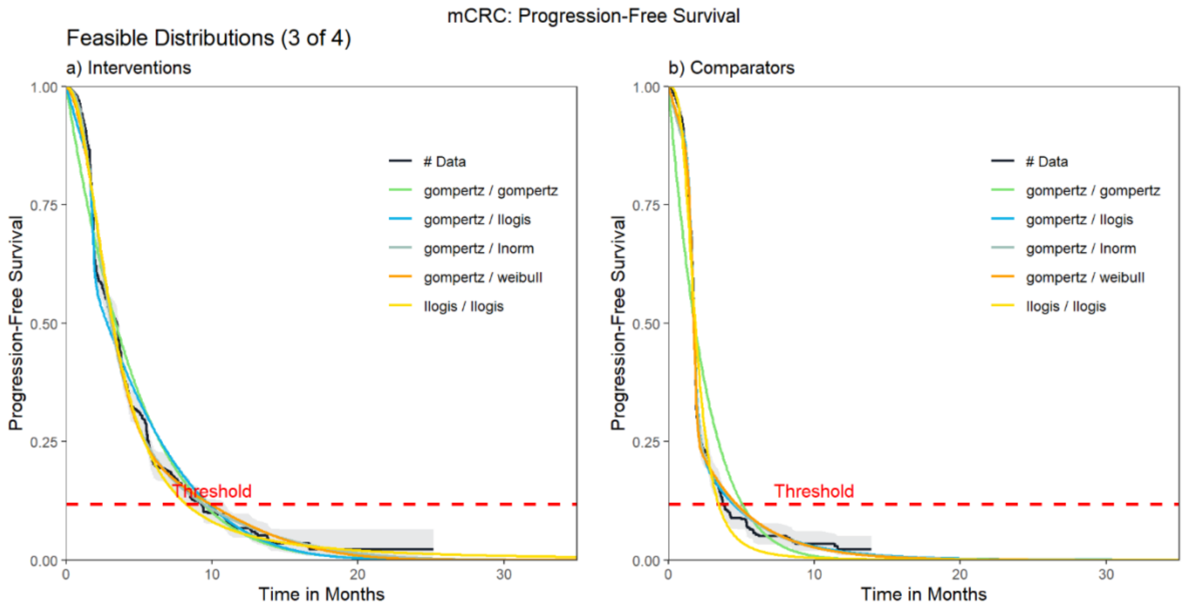

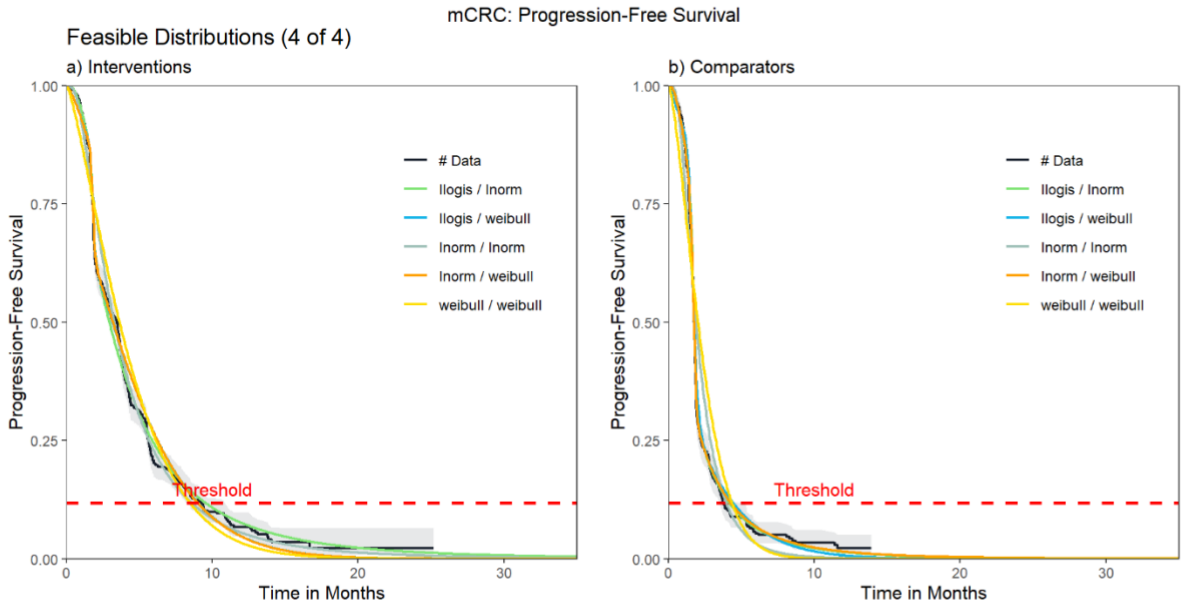

3.2.3 Infeasible Extrapolations

There were no infeasible extrapolations for metastatic colorectal cancer.

3.2.4 Selected Extrapolations

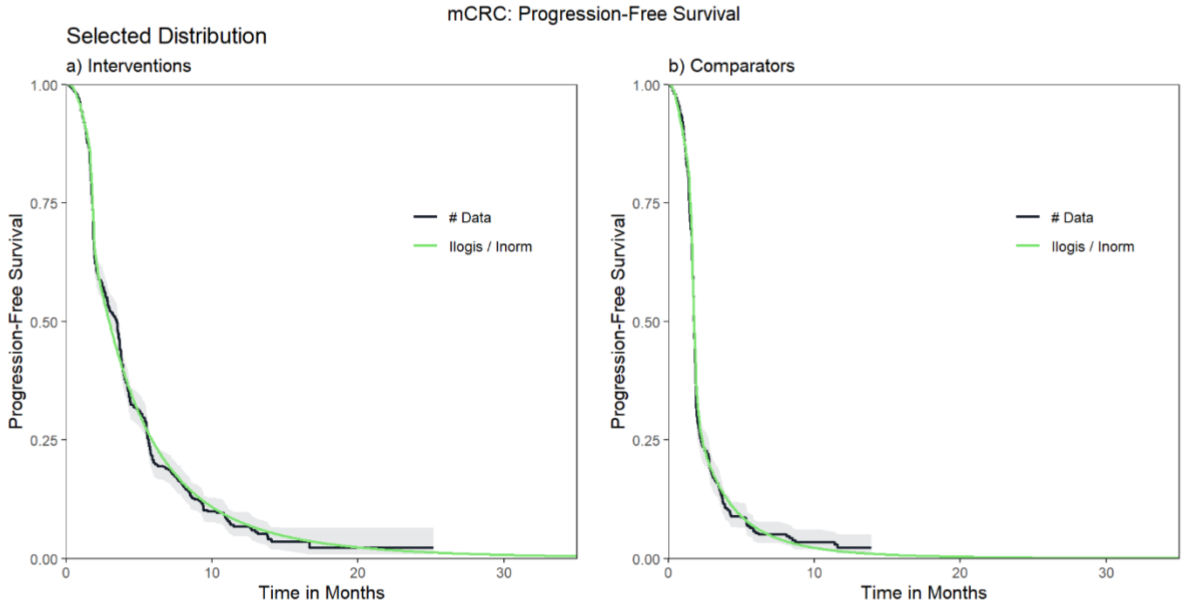

3.3 Metastatic Non-Small Cell Lung Cancer (mNSCLC)

3.3.1 Survival Extrapolation Selection process

		INTERVENTION					COMPARATOR					COMBINED	
	**Distribution**	**pFeasible**	**Feasible?**	**nPar**	**LogLik**	**AIC**	**pFeasible**	**Feasible?**	**nPar**	**LogLik**	**AIC**	**AIC**	**Rank**
1	gamma	5.87E-05	Yes	2	-2735.26	5474.515	8.43E-08	Yes	2	-2703.09	5410.178	10884.69	14
2	gompertz	0.101587	No	NA	NA	NA	1.99E-10	Yes	2	-2716.91	5437.813	NA	NA
3	llogis	0.024547	Yes	2	-2680.52	5365.047	0.008661	Yes	2	-2699.37	5402.741	10767.79	11
4	lnorm	0.014275	Yes	2	-2659.92	5323.848	0.002677	Yes	2	-2691.49	5386.986	10710.83	9
5	weibull	0.000234	Yes	2	-2730.36	5464.725	1.23E-08	Yes	2	-2707.98	5419.953	10884.68	13
6	gamma / gamma	5.87E-05	Yes	5	-2735.26	5480.515	8.43E-08	Yes	5	-2703.09	5416.178	10896.69	16
7	gamma / gompertz	0.036047	Yes	5	-2613.77	5237.533	2.16E-13	Yes	5	-2693.39	5396.785	10634.32	6
8	gamma / llogis	8.81E-05	Yes	5	-2603.82	5217.63	6.09E-08	Yes	5	-2632.74	5275.481	10493.11	1
9	gamma / lnorm	0.00012	Yes	5	-2614.45	5238.907	5.96E-08	Yes	5	-2632.31	5274.624	10513.53	4
10	gamma / weibull	0.00015	Yes	5	-2619.01	5248.029	1.36E-10	Yes	5	-2692.39	5394.782	10642.81	7
11	gompertz / gompertz	0.10161	No	NA	NA	NA	1.99E-10	Yes	5	-2716.91	5443.813	NA	NA
12	gompertz / llogis	0.053486	No	NA	NA	NA	0.005265	Yes	5	-2694.89	5399.777	NA	NA
13	gompertz / lnorm	0.053059	No	NA	NA	NA	0.001694	Yes	5	-2687.71	5385.427	NA	NA
14	gompertz / weibull	0.02619	Yes	5	-2627.71	5265.42	0	Yes	5	-2696.35	5402.703	10668.12	8
15	llogis / llogis	0.024552	Yes	5	-2680.52	5371.047	0.008661	Yes	5	-2699.37	5408.741	10779.79	12
16	llogis / lnorm	0.016633	Yes	5	-2563.5	5137.003	0.002801	Yes	5	-2677.41	5364.818	10501.82	2
17	llogis / weibull	0.000172	Yes	5	-2603.87	5217.734	7.36E-10	Yes	5	-2637.66	5285.313	10503.05	3
18	lnorm / lnorm	0.014275	Yes	5	-2659.92	5329.848	0.002677	Yes	5	-2691.49	5392.986	10722.83	10
19	lnorm / weibull	0.000151	Yes	5	-2615.72	5241.446	0.001387	Yes	5	-2685.22	5380.443	10621.89	5
20	weibull / weibull	0.000234	Yes	5	-2730.36	5470.725	1.23E-08	Yes	5	-2707.98	5425.953	10896.68	15

Table 6: Survival Extrapolation Selection Process for Progression-Free Survival (PFS) - Metastatic Non-Small Cell Lung Cancer (mNSCLC)

3.3.2 Feasible Extrapolations

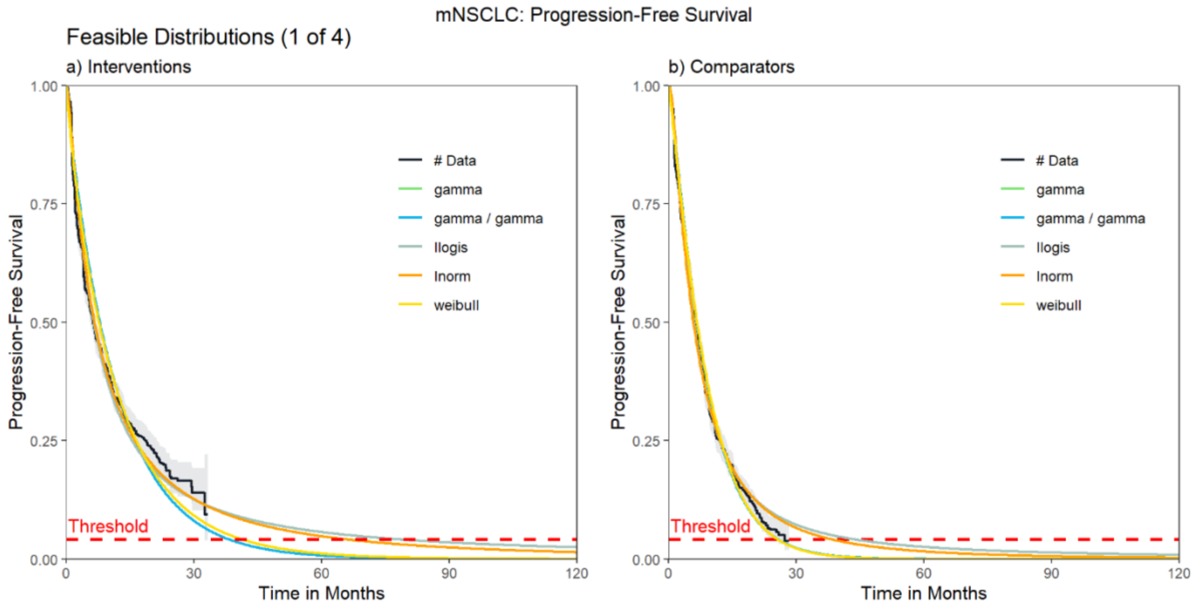

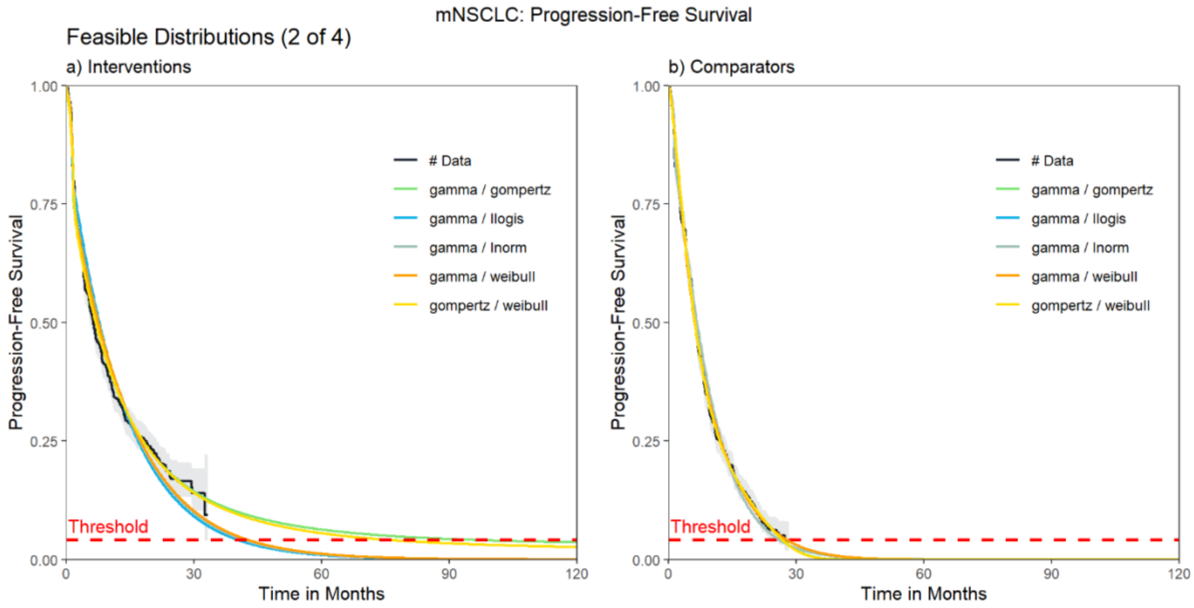

Note: Even though the gamma / llogis distribution was ranked number 1, it substantially underestimated survival for the interventions, and therefore was not selected.

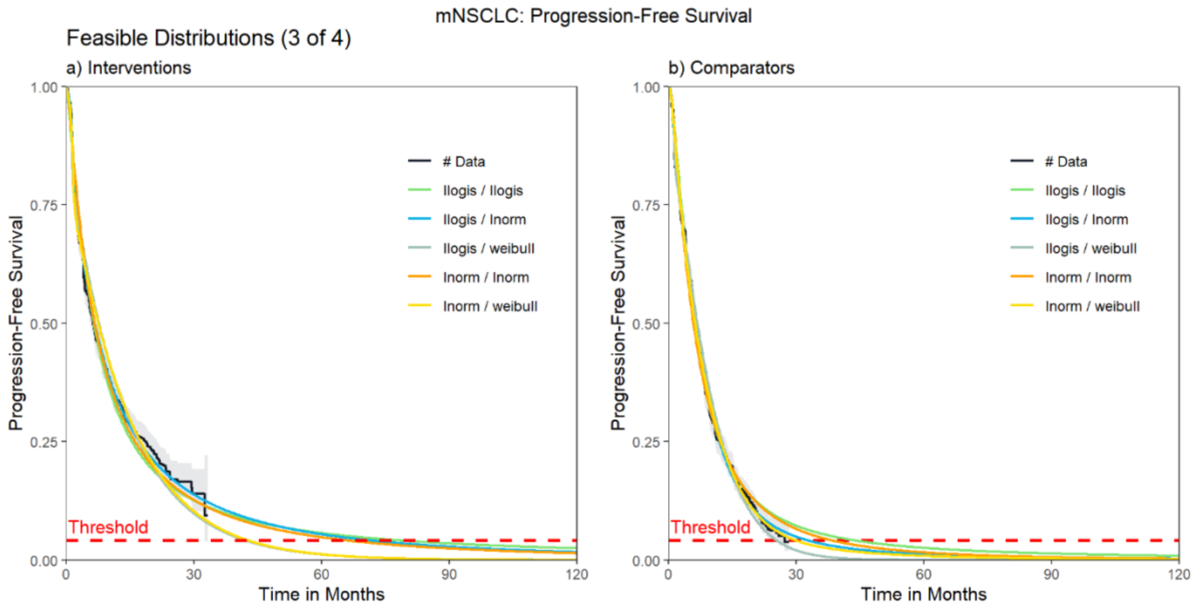
mNSCLC: Progression-Free Survival
Feasible Distributions (3 of 4)
a) Interventions
b) Comparators
Progression-Free Survival
Time in Months
Data
llogis / llogis
llogis / lnorm
llogis / weibull
lnorm / lnorm
lnorm / weibull
Threshold
1.00
0.75
0.50
0.25
0.00
0
30
60
90
120

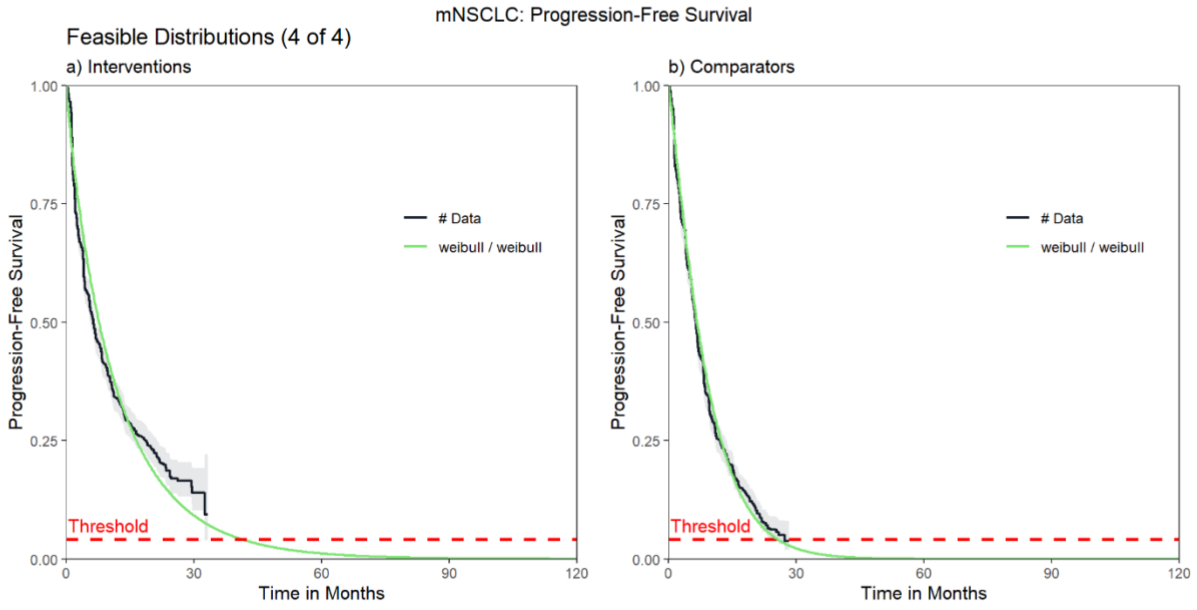
mNSCLC: Progression-Free Survival
Feasible Distributions (4 of 4)
a) Interventions
b) Comparators
Progression-Free Survival
Time in Months
Data
weibull / weibull
Threshold
1.00
0.75
0.50
0.25
0.00
0
30
60
90
120

3.3.3 Infeasible Extrapolations

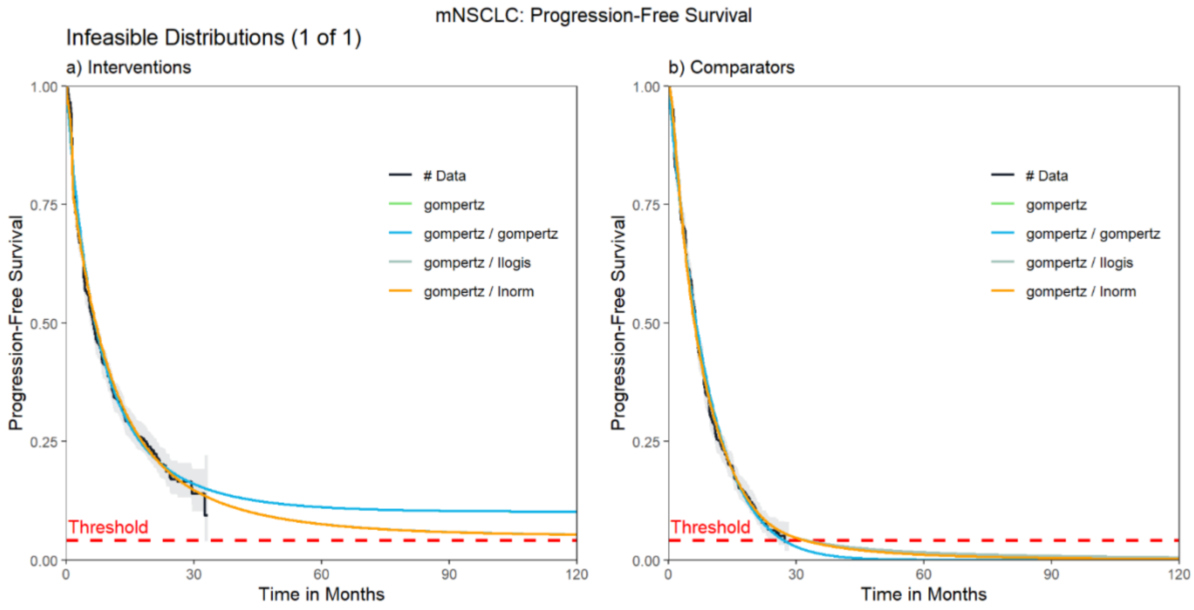

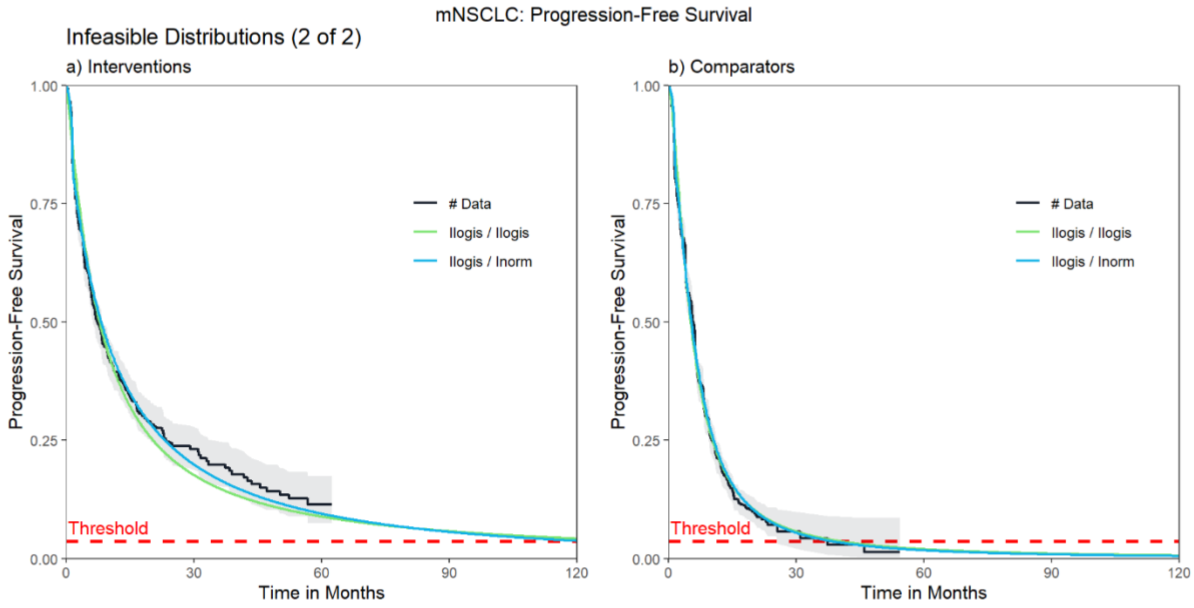

3.3.4 Selected Extrapolations

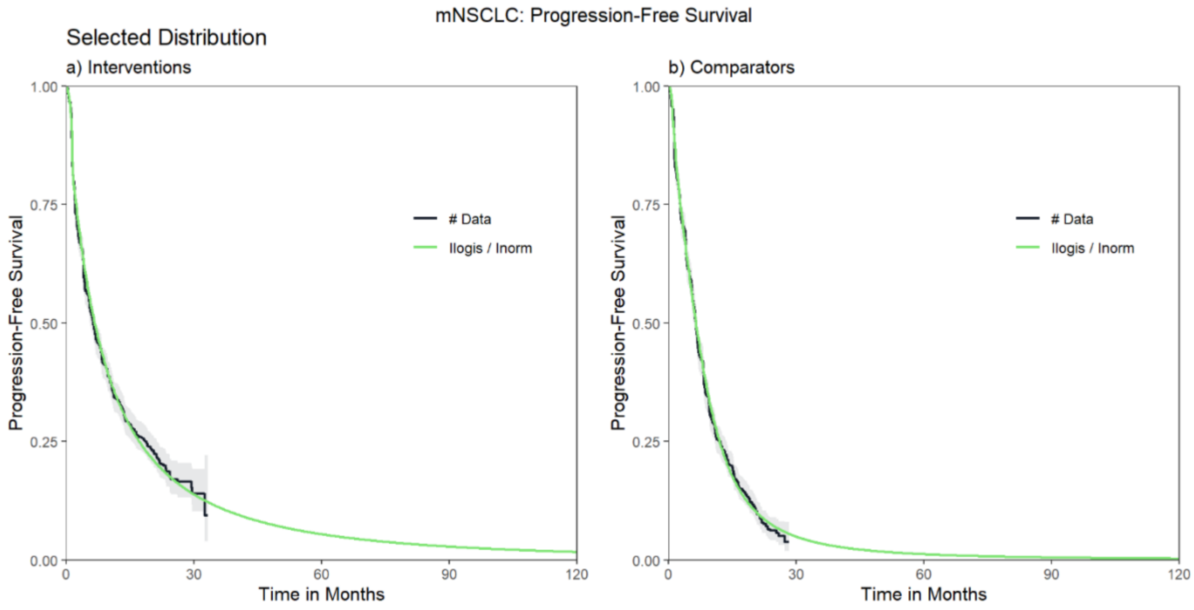

Supplement: Supplementary file 1 [file DataSheet1.pdf]
